# Microbial Metagenomes and Host Transcriptomes Reveal the Dynamic Changes of Rumen Gene Expression, Microbial Colonization and Co-Regulation of Mineral Element Metabolism in Yaks from Birth to Adulthood

**DOI:** 10.3390/ani14091365

**Published:** 2024-04-30

**Authors:** Yili Liu, Liangliang Ma, Daojie Riqing, Jiu Qu, Jiyong Chen, Danzeng Zhandu, Biao Li, Mingfeng Jiang

**Affiliations:** 1 Key Laboratory of Qinghai-Tibetan Plateau Animal Genetic Resource Reservation, College of Animal & Veterinary Sciences, Southwest Minzu University, Chengdu 610041, China; yililiunum@163.com (Y.L.); 19822966564@163.com (D.R.); 18874028597@163.com (B.L.); 2 College of Grassland Resources, Southwest Minzu University, Chengdu 610041, China; maliangliang2020@163.com; 3 Agriculture and Rural Affairs Bureau of Naqu City, Naqu 852000, China; xnmz2024@126.com (J.Q.); 18908968800@163.com (D.Z.); 4 Yushu Prefecture Animal Disease Prevention and Control Center, Yushu 815000, China; yschjy815000@163.com

**Keywords:** yaks, rumen, age, transcriptome, metagenomic, ion binding

## Abstract

**Simple Summary:**

Minerals are essential for maintaining the health and productivity of livestock. Yaks are mainly grazed rotationally in the Qinghai-Tibet Plateau, but the natural forage nutrients in the alpine meadow are seasonal and even different every month, and the nutritional requirements of yaks at different ages are different. Therefore, it is very important to understand how host genes and microorganisms in the rumen of yaks at different ages coordinate to regulate the metabolism of mineral elements and other nutrients. In this study, we performed transcriptome and metagenomic analyses of the rumen and its contents at different age stages to explore the synergistic regulation of mineral metabolism by host genes and microorganisms. In total, 13 bacteria and 22 host genes were mainly involved in the regulation of metal ion binding. The results of this study are of great significance for the improvement of supplementary feeding and growth performance of yaks at different ages.

**Abstract:**

Yaks are the main pillar of plateau animal husbandry and the material basis of local herdsmen’s survival. The level of mineral elements in the body is closely related to the production performance of yaks. In this study, we performed a comprehensive analysis of rumen epithelial morphology, transcriptomics and metagenomics to explore the dynamics of rumen functions, microbial colonization and functional interactions in yaks from birth to adulthood. Bacteria, eukaryotes, archaea and viruses colonized the rumen of yaks from birth to adulthood, with bacteria being the majority. Bacteroidetes and Firmicutes were the dominant phyla in five developmental stages, and the abundance of genus *Lactobacillus* and *Fusobacterium* significantly decreased with age. Glycoside hydrolase (GH) genes were the most highly represented in five different developmental stages, followed by glycosyltransferases (GTs) and carbohydrate-binding modules (CBMs), where the proportion of genes coding for CBMs increased with age. Integrating host transcriptome and microbial metagenome revealed 30 gene modules related to age, muscle layer thickness, nipple length and width of yaks. Among these, the MEmagenta and MEturquoise were positively correlated with these phenotypic traits. Twenty-two host genes involved in transcriptional regulation related to metal ion binding (including potassium, sodium, calcium, zinc, iron) were positively correlated with a rumen bacterial cluster 1 composed of *Alloprevotella*, *Paludibacter*, *Arcobacter*, *Lactobacillus*, *Bilophila*, etc. Therefore, these studies help us to understand the interaction between rumen host and microorganisms in yaks at different ages, and further provide a reliable theoretical basis for the development of feed and mineral element supplementation for yaks at different ages.

## 1. Introduction

The yak (*Bos grunniens*) is a rare and primitive ruminant, which is mainly distributed on the Qinghai–Tibetan Plateau (high, cold, low oxygen and strong ultraviolet rays) and adjacent high mountainous areas with an altitude above 3000 m [1]. Maiwa yaks are mainly produced in these areas of Wache, Maiwa in Hongyuan County, and Baozuo of Ruoergai County, Aba Tibetan Autonomous Prefecture. Throughout the year, yaks primarily rely on natural forages for nutrition, while during the long cold season, they are seriously challenged by poor foraging resources [2,3]. The yaks grow with a vicious seasonal cycle of strong in summer, fat in autumn, thin in winter and tired in spring, which is mainly affected by the seasonal variations in the temperature and quantity of natural herbage [4,5]. This also leads to the low production efficiency of yaks, and seriously restricts the utilization of germplasm characteristics and industrial development.

The rumen is a unique digestive organ of ruminants. From birth to adulthood, rumen structure and function change dramatically [6]. Newborn ruminants mainly consume liquid milk through the abomasum for energy supply, while the rumen is the main place for nutrient digestion and absorption in adult ruminants [7]. The rumen contains a variety of microorganisms that are responsible for converting low-quality forage into high-quality animal proteins that can be absorbed by the host through microbial fermentation [8,9]. Those microbes work together synergistically and contribute substantially to the nutrient utilization, metabolism, growth performance and health of the host [10,11]. In this process, in addition to the role of the gastrointestinal symbiotic microbiome, the host genome itself also plays a key role [12]. The host and microbial genomes must coordinate their work and perform their respective functions to maintain their body health under various environmental conditions [13]. Many factors, such as age, diet, breed and geographical location, have direct or indirect effects on the development of the whole rumen [14,15], among which age is a direct influencing factor in shaping gut microbiome. The transcriptome and microbiome profiles of the rumen vary with different developmental stages [16]. In the development process of ruminants such as Simmental beef cattle, dairy Murrah buffaloes [17] and goats [18], the effects of age on microbial communities and host physiological responses have also been reported. Transcriptome analysis of Simmental half-sib individuals found that rumen development and beef traits are closely related to developmental stages [16]. The composition and function of rumen microbiota in dairy Murrah buffaloes (from 1 year to more than 9 years old) change significantly with age, and the proportion of Bacteroides and Methanobrevibacter increases significantly with age, while the *Lactobacillus* shows the opposite trend [18]. The development and maturity of rumen microbiota (bacteria, archaea, protozoa and fungi) in grazing yaks are closely related to age, with archaea reaching full maturation at about 5 years old and the other microbiota reaching maturity between 5 and 8 years of age [19]. Moreover, age-related microbial changes are strongly associated with inflammaging [20] and methane production [21]. However, there are few combined omics analyses of the rumen at different ages. The rumen as a whole, the host itself and the types and metabolism of microorganisms work in coordination with each other, and studies on a single aspect cannot better reflect the regulatory mechanism of rumen on the whole body. 

More studies have shown that it is important to reveal the relevant laws and mechanisms of host physiological characteristics by comprehensively sequencing the gastrointestinal microbiome and metagenome, and correlating these data with the host genome, transcriptome and metabolism. The interactions among the rumen transcriptome, microRNAome, and microbial metagenome in Holstein bull calves suggest that a highly active early microbiome can regulate the rumen development of neonatal calves, and miRNAs may coordinate these host–microbial interactions [22]. Multi-omics analysis of rumen transcriptome, microbiome and metabolome of Tibetan sheep shows that they jointly act on some key pathways related to cold season adaptation, explaining the molecular and metabolic mechanisms of Tibetan sheep during the cold season [23]. Integrated transcriptome and microbiome analyses show that nutritional intervention can improve rumen functions and promote compensatory growth of stunted yaks [24]. The combined analysis of the microbiome and metabolomics reveals that different feeding systems significantly change the abundance of microbiota and affect the concentration and metabolic pathways of rumen-related metabolites, thereby jointly regulating rumen growth and development in yaks [25]. However, information on the effects of different ages on rumen microbiota and host transcriptome interactions is still lacking, especially in yaks. To our knowledge, there are few studies that combine rumen microbiome and host transcriptome to jointly analyze the effects of age on rumen growth, development and metabolism, focusing mainly on the limited stages of Holstein bull calves (weeks 1, 3, 6) [22] and goats (days 1, 7, 14, 21, 28, 42 and 56) [26].

To fill this gap and further explore the effect of age on the functional correlation between rumen host and their microbiota, rumen tissue and fluid samples from 28 yaks at 5 different time points were sequenced. This study was based on three stages of rumen tissue morphological development: non-rumination stage (0–3 weeks), transitional stage (3–8 weeks) and rumination stage (after 8 weeks) [27]. Grazing yaks of 1 day old, 20 days old, 60 days old, 15 months old and 3 years old were selected as research objects, and transcriptome and metagenomic analysis were performed on rumen tissue and rumen fluid, respectively. Combining with histomorphological identification, the rumen of yaks at different developmental stages was studied in order to provide theoretical reference and new ideas for the supplementary feeding of yaks of different ages.

## 2. Materials and Methods

### 2.1. Ethics Statement

All experimental procedures involving animals were reviewed and approved by the Institutional Animal Care and Use Committee at Southwest Minzu University (Chengdu, Sichuan, China), and all studies were in line with the requirements of the directory of the Ethical Treatment of Experimental Animals of China.

### 2.2. Experimental Design and Sample Collection

**Figure 1:**
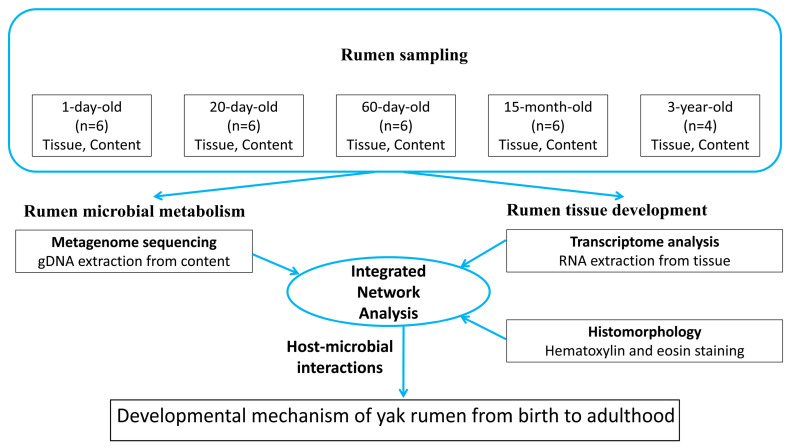
Schematic diagram of test design.

The Maiwa yaks in Hongyuan County, Aba Tibetan and Qiang Autonomous Prefecture, Sichuan Province, were taken as the research object, with an altitude of 3500 m. All yaks were grazed naturally without feed supplementation, and fed with natural milk and pasture. Twenty-eight healthy yaks were divided into five groups based on ages: 1 d (1 day old, n = 6), 20 d (20 days old, n = 6), 60 d (60 days old, n = 6), 15 m (15 months old, n = 6), and 3 y (3 years old, n = 4), Each group was composed of healthy yaks of similar weight. Due to the large size of yaks, it is not suitable to use anesthetic during the sampling process, so a more extensive and humane method of electric shock was adopted in the experiment. To ameliorate suffering, yaks were humanely sacrificed according to the following procedures: electrically stunned (120 V dc, 12 s) before exsanguination, sacrificed while in the coma by bloodletting from carotid artery and jugular vein, and dissected rapidly to obtain the whole rumen from each individual. The collected rumen samples (tissue and content separately) were quickly frozen in liquid nitrogen tanks for subsequent transcriptome analysis and metagenome sequencing. The experimental design is shown in Figure 1.

### 2.3. Morphological Characteristics

The 1 cm × 1 cm × 0.5 cm rumen tissues from five groups were collected and fixed in 10% neutral formaldehyde fixative for 24 h, followed by dehydration, transparency, waxing, embedding, slicing and dyeing. Hematoxylin and eosin were used for staining (H.E). Hematoxylin stained the nucleus blue-purple, and eosin stained the cytoplasm pink. All slides were observed under a microscope BA410Digital (Motic China Group Co., Ltd., Xiamen, China). Images were analyzed and acquired using the Image analysis software (Motic Images Advanced 3.2). The thickness of muscularis, papillary height and papillary width were determined using CaseViewer section analysis system.

### 2.4. RNA Extraction, Transcriptome Analysis and Reverse Transcription-Quantitative PCR Verifification

The mirVana miRNA Isolation Kit (Ambion-1561, Austin, TX, USA) was used to extract the total RNA from the rumen tissue of yaks. The Nanodrop 2000 (Thermo Fisher Scientific, Waltham, MA, USA) was used for RNA concentration determination, and the Agilent 2100 Bioanalyzer (Agilent Technologies, Santa Clara, CA, USA) for evaluating the integrity. The samples with RNA Integrity Number (RIN) ≥ 7 were subjected to the subsequent analysis.

The cDNA Library was constructed with a TruSeq Stranded mRNA LTSample Prep Kit (Illumina, San Diego, CA, USA), and the manufacturer’s protocol was strictly followed. These libraries were purified using Agencourt AMPure XP beads (Beckman Coulter, Inc., Brea, CA, USA) and further analyzed for integrity, purity and size with an Agilent 2100 Bioanalyzer (Agilent Technologies, Santa Clara, CA, USA). Libraries were sequenced on the Illumina sequencing platform (Illumina HiSeq X Ten) by Shanghai OE Biotech Co., Ltd. (Shanghai, China). Raw reads were processed using Trimmomatic software. Clean data were obtained after data filtering of raw reads containing ploy-N and the low-quality reads. Then the clean reads were mapped to the yak reference genome (GCF_000298355.1) using hisat2 [28]. The FPKM value of each gene was calculated by cufflinks [29,30], and the number of reads mapped to each gene was counted by htseq-count [31]. FPKM was used to measure the transcription or gene expression level. Differentially expressed genes (DEGs) were identified using the DESeq R package functions estimateSizeFactors and nbinomTest [32]. The fold change (FC) represented the ratio of expression levels between two groups, and adjusted *p* value < 0.05 and fold Change ≥ 2 or fold Change ≤ 0.5 was set as the threshold for significantly differential expression. Hierarchical cluster analysis of DEGs was performed to explore gene and transcript expression patterns. GO enrichment [33] and KEGG (Kyoto Encyclopedia of Genes and Genomes) [34] pathway enrichment analysis of DEGs were performed using R based on the hypergeometric distribution. Weighted gene co-expression network analysis (WGCNA) [35] was performed to understand the link between the host transcriptome and the phenotypic traits (age, muscle layer thickness, papillae length and papillae width).

In order to verify the reliability and reproducibility of RNA-seq data, five DEGs were randomly selected for RT-qPCR quantification. The total RNA was extracted using the mirVana miRNA Isolation Kit (Ambion, Austin, TX, USA). cDNA was synthesized by reverse transcription using the PrimeScript RT reagent Kit with gDNA Eraser (TaKaRa, Dalian, China). RT-qPCR was performed using the LightCycler 96 System (Roche Diagnostics, Indianapolis, IN, USA) according to the manufacturer’s instructions. The forward and reverse primers of these DEGs are shown in Supplemental Table S1. Three biological replicates were analyzed per sample. The gene validation for each time point was performed in triplicate. The expression level of each validated gene for each time point was calculated by the 2^−∆∆Ct^ method.

### 2.5. Metagenomic Sequencing and Bioinformatics Analysis

Genomic DNA of the rumen content samples was extracted using the QIAamp DNA Stool Kit (Qiagen, Hilden, Germany). DNA concentration was then measured using the Qubit^®^ dsDNA Assay Kit in Qubit^®^ 2.0 Fluorometer (Life Technologies, Carlsbad, CA, USA). For each sample, an OD value between 1.8~2.0 and DNA contents above 1 μg can be used to construct the library. The NEBNext^®^ Ultra™ DNA Library Prep Kit (Illumina, NEB, San Diego, CA, USA) was applied to perform metagenome libraries construction according to the manufacturer’s protocol. Metagenomic sequencing was performed on an Illumina HiSeq PE150 platform by the Novogene Co., Ltd. (Beijing, China), and the raw data was obtained. 

The raw data were preprocessed using Readfq (V8, https://github.com/cjfields/readfq, accessed on 29 April 2024) and the clean data were acquired for subsequent analysis. The clean data were assembled and analyzed by SOAPdenovo software (V2.04, http://soap.genomics.org.cn/soapdenovo.html, accessed on 29 April 2024). All the reads not used in the forward step of all samples were combined, and then the SOAPdenovo software was used for mixed assembly. In all the scaftigs, fragments shorter than 500 bp obtained from the single or mixed assembly were filtered for further statistical analysis. All scaftigs (≥500 bp) were used to predict open reading frames (ORFs) using MetaGeneMark software (V2.10, http://topaz.gatech.edu/GeneMark/, accessed on 29 April 2024). For predicted ORF, CD-HIT software (V4.5.8) [36,37] was used to remove redundant ORFs, and the unique initial gene catalogue was obtained. The clean data of each sample were mapped to the initial gene catalogue using Bowtie 2.2.4 software (http://bowtie-bio.sourceforge.net/bowtie2/index.shtml, accessed on 29 April 2024). DIAMOND software (V0.9.9) [38] was adopted to blast the unigenes to the sequences of bacteria, fungi, archaea and viruses which were all extracted from the NR database of NCBI. Then, we adopted the DIAMOND software to blast and annotate unigenes to functional databases including the KEGG, eggNOG (Non-supervised Orthologous Groups), and CAZy database. 

### 2.6. Data Analysis

All experiments results had at least three replicates and were expressed as mean ± standard deviation (S.D.). Statistical evaluation was performed using ANOVA (SPSS 18.0), and *p* values < 0.05 were considered significant. Spearman’s correlation test (*p* < 0.05, r > 0.6) was performed to analyze the correlation between level 2 microbial functions and host genes involved in rumen epithelial tissue development.

## 3. Results 

### 3.1. Morphological Analysis of Rumen Epithelium 

**Figure 2:**
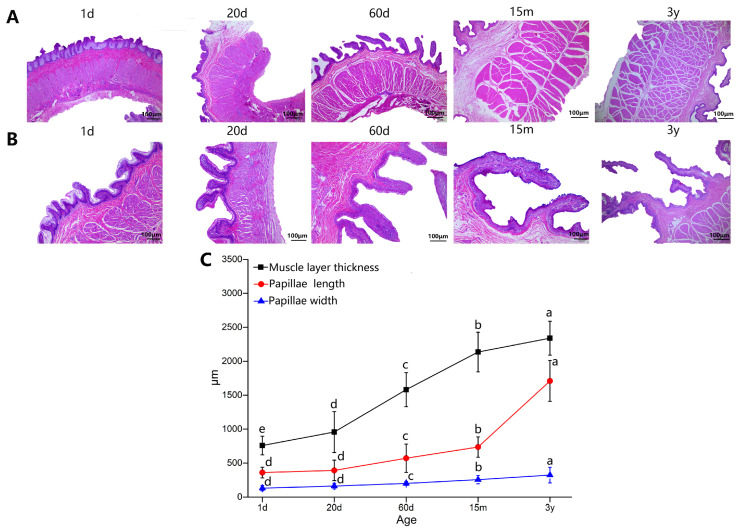
Morphology of rumen epithelial tissue of yaks in five developmental stages. (**A**) Muscle layer thickness. (**B**) Papillae length and papillae width. Images are obtained through a light micrograph of rumen tissue at a magnification of ×4 (**A**) and ×10 (**B**) objective lens (bar = 100 μm). (**C**) Measurement of muscle layer thickness, papillae length and width. In the same curve, the difference between different small letters in the corner mark is significant (*p* < 0.05), and the same letter indicates that the difference is not significant (*p* > 0.05).

The morphology and three measured indicators of rumen are shown in Figure 2. Rumen muscle fibers were densely arranged and papillae were closely arranged at 1 day and 20 days. Compared with 1 day, rumen papillae length (PL) and width (PW) at 20 days increased, but not significantly. At 60 days, the rumen muscle fibers were spaced, and the PL and PW increased significantly. At 15 months and 3 years, the muscle fiber gap of rumen was more obvious, and the PL and PW further increased (Figure 2A,B). The statistical results showed that the muscle layer thickness of rumen increased markedly with age (*p* < 0.05). There were no significant differences in PL and PW between yaks at 1 and 20 days (*p* > 0.05). After 20 days, the PL and PW significantly increased with age (*p* < 0.05) (Figure 2C). The morphological changes of slices were basically consistent with the statistical results.

### 3.2. Transcriptional Profile in the Rumen Epithelial Tissue

#### 3.2.1. Analysis of Differentially Expressed Genes 

**Figure 3:**
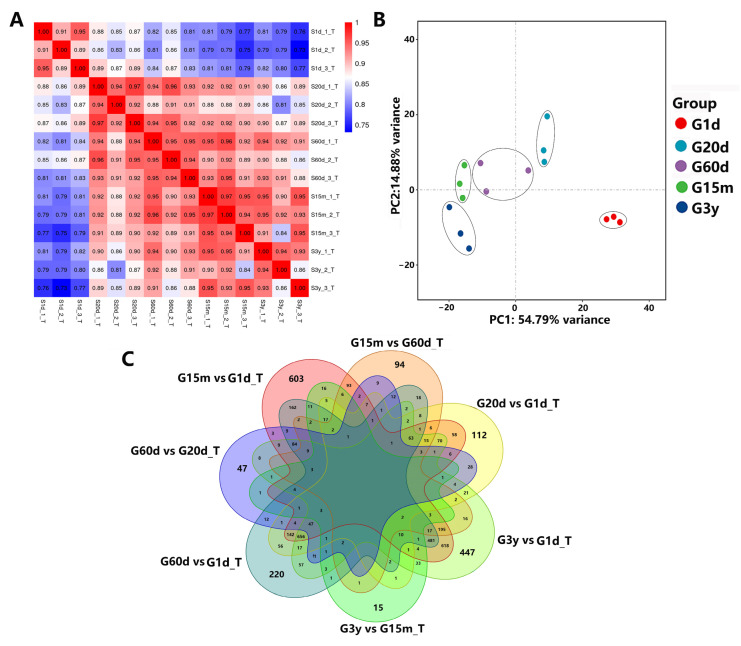
Difference analysis of rumen epithelial transcriptome in five developmental stages. (**A**) Heat map analysis of correlation between individual tissue samples. (**B**) Principal component analysis (PCA) of gene expressions in the rumen tissue among five groups. (**C**) Venn diagram analysis of all expressed genes among seven comparison groups.

Transcriptome sequencing analysis was performed on the rumen epithelial tissue of yaks across five developmental stages (1 d, 20 d, 60 d, 15 m and 3 y after birth), and 101.57 Gb clean data were obtained. The clean data from each sample reached 6.08~7.27 Gb, and the percentage of Q30 bases was 93.36% and above. The correlation diagram showed that the correlation values within five developmental stages were greater than 0.89, 0.92, 0.94, 0.94 and 0.86, respectively (Figure 3A). PCA showed that there was a significant difference among five developmental stages (*p* < 0.05), and the repeatability within each group was relatively good (Figure 3B). 

**Figure 4:**
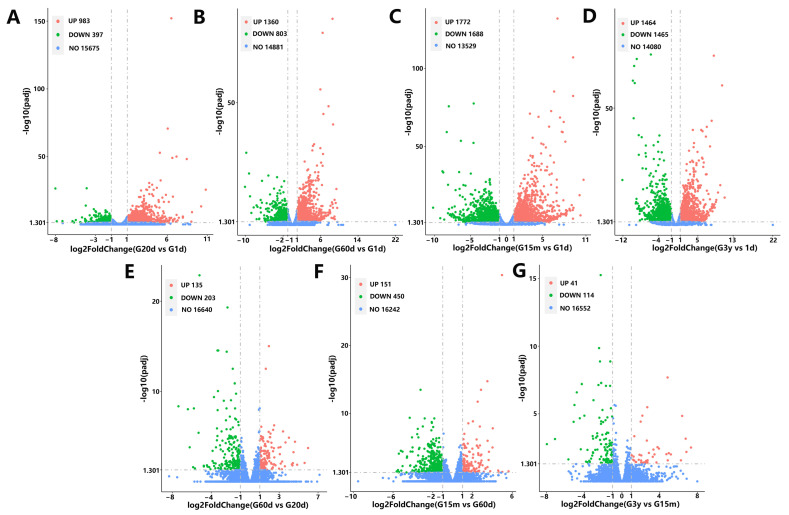
The volcano plot of differently expressed genes in seven comparison groups. Padj ≤ 0.05 |log2FoldChange| ≥ 1.0. (**A**) 20 days vs. 1 days (20 d vs. 1 d). (**B**) 60 days vs. 1 day (60 d vs. 1 d). (**C**) 15 months vs. 1 day (15 m vs. 1 d). (**D**) 3 years vs. 1 day (3 y vs. 1 d). (**E**) 60 days vs. 20 days (60 d vs. 20 d). (**F**) 15 months vs. 60 days (15 m vs. 60 d). (**G**) 3 years vs. 15 months (3 y vs. 15 m).

To explore common DEGs during the five developmental stages, four closed groups (20 d vs. 1 d, 60 d vs. 1 d, 15 m vs. 1 d and 3 y vs. 1 d) and three consecutive groups (60 d vs. 20 d, 15 m vs. 60 d, and 3 y vs. 15 m) were designed to construct Venn diagrams. In the seven comparison groups, common DEmRNAs were not detected, and the unique genes observed were 112, 220, 603, 447, 47, 94 and 15, respectively (Figure 3C). A total of 1380, 2163, 3460, 2929, 338, 601 and 155 DEGs were found in the seven comparison groups, of which 983, 1360, 1772, 1464, 135, 151 and 41 were upregulated, and 397, 803, 1688, 1465, 203, 450 and 114 were downregulated (Figure 4). In four closed groups, compared with 1 d, the top 5 expressed genes were *A2ML1*, *S100A12*, *LOC102283503*, *S100A9* and *KRT6A* at 20 d (Supplemental Table S2), *A2ML1*, *LOC102283503*, *LOC102266213*, *KRT6A* and *LOC102271013* at 60 d (Supplemental Table S3), *A2ML1*, *LOC102283503*, *DSP*, *KRT6A* and *LOC102266213* at 15 m (Supplemental Table S4), *LOC102264614*, *KRT6A*, *LOC102266213*, *LOC102283503* and *LOC102268548* at 3 y (Supplemental Table S5). In three consecutive groups, the five genes with the highest expression were *LOC102266213*, *MFGE8*, *POSTN*, *ANXA1* and *S100A9* in the 60 d vs. 20 d group (Supplemental Table S6), *TGM3*, *COL3A1*, *COL1A1*, *SPARC* and *COL1A2* in the 15 m vs. 60 d group (Supplemental Table S7), *LOC102284246*, *KRT78*, *LOC106701036*, *ENDOD1* and *LOC102282831* in the 3 y vs. 15 m group (Supplemental Table S8). In addition, in the 20 d vs. 1 d group, the upregulated genes with FC > 140 and FPKM > 20 were screened: *LOC102285929*, *KLK14*, *LOC102272875*, *LOC102283173*, *LOC102281207*, *CD4*, *NAPSA* and *LOC102271453*, where *LOC102285929*, *KLK14*, *LOC102272875* and *LOC102283173* significantly increased by 1811, 1211, 549 and 518 times, respectively (Supplemental Table S2). 

#### 3.2.2. Functional Enrichment Analysis of Differentially Expressed Genes in Rumen Functional Transformation

To study the role of DEGs in the transformation of rumen function, the significantly enriched GO terms and the top 20 KEGG enrichment pathways in four consecutive groups were analyzed in Supplemental Figures S1 and S2, Tables S9 and S10. In the GO database, annotated genes are classified into three categories: biological processes (BP), molecular functions (MF), and cell components (CC). As shown in Supplemental Figure S1 and Table S9, in the 20 d vs. 1 d group, the most significantly enriched GO terms of DEGs were defense response to other organism (GO:0098542), mitotic nuclear division (GO:0140014), response to bacterium (GO:0009617), glycoside metabolic process (GO:0016137) and polyketide metabolic process (GO:0030638). In the 60 d vs. 20 d group, the most significantly enriched GO terms of DEGs were concentrated in active transmembrane transporter activity (GO:0022804), apical part of cell (GO:0045177), apical plasma membrane (GO:0016324), keratinocyte differentiation (GO:0030216) and epidermal cell differentiation (GO:0009913). In the 15 m vs. 60 d group, the most significantly enriched GO terms were extracellular matrix (GO:0031012), extracellular matrix structural constituent (GO:0005201), extracellular matrix component (GO:0044420), proteinaceous extracellular matrix (GO:0005578) and extracellular structure organization (GO:0043062). In the 3 y vs. 15 m group, the most significantly enriched GO terms were involved in humoral immune response (GO:0006959), humoral immune response mediated by circulating immunoglobulin (GO:0002455), complement activation, classical pathway (GO:0006958), metal ion transmembrane transporter activity (GO:0046873) and immunoglobulin-mediated immune response (GO:0016064).

KEGG enrichment analysis of the DEGs in four consecutive groups were performed, and the results are shown in Supplemental Figure S2 and Table S10. In the four comparison groups, there were 40, 6, 8 and 5 significantly upregulated signaling pathways, and 1, 0, 11 and 6 markedly downregulated signaling pathways, respectively (*p* < 0.05). Compared to 1 d, the dramatically upregulated DEGs at 20 d were mainly associated with immune response, metabolism and growth and development related pathways, such as hematopoietic cell lineage, primary immunodeficiency, intestinal immune network for IgA production, steroid biosynthesis, pentose and glucuronate interconversions, butanoate metabolism, arachidonic acid metabolism and cell cycle, and downregulated pathways were MAPK signaling pathways. Compared to 20 d, the significantly upregulated DEGs at 60 d were primarily involved in such metabolic and immune pathways as drug metabolism-cytochrome P450, retinol metabolism, steroid hormone biosynthesis, metabolism of xenobiotics by cytochrome P450, African trypanosomiasis and legionellosis, but no significant downregulated signaling pathway was observed. In addition, between 15 m and 60 d, considerably upregulated pathways were all related to immune stress and amino acid metabolic pathways, including chemical carcinogenesis, the IL-17 signaling pathway, ferroptosis, drug metabolism-cytochrome P450, the TNF signaling pathway, metabolism of xenobiotics by cytochrome, P450 phenylalanine metabolism and histidine metabolism, and downregulated genes were primarily enriched in protein digestion and absorption, ECM-receptor interaction, axon guidance, focal adhesion, the PI3K-Akt signaling pathway and other pathways. Compared to 15 m, upregulated genes at 3 y were significantly enriched in drug metabolism-cytochrome P450, metabolism of xenobiotics by cytochrome P450, retinol metabolism, chemical carcinogenesis and cortisol synthesis and secretion, and all significant downregulated pathways were closely related to immune response, such as systemic lupus erythematosus, staphylococcus aureus infection, complement and coagulation cascades, etc.

#### 3.2.3. RT-qPCR Quantification of mRNAs

Five mRNAs—*BDH1*, *ECHS1*, *FDPS*, *HMGCS2* and *PCCA*—were randomly selected for reliable verification of RNA-seq data in yaks. The selected genes were quantitatively analyzed by RT-qPCR at five developmental stages. The results showed that RT-qPCR data were consistent with RNA-seq data, indicating that RNA-seq data were reliable (Supplemental Figure S3). 

### 3.3. Profiling of the Rumen Metagenome

#### 3.3.1. Comparison of Rumen Microbiome across Different Age Groups

**Figure 5:**
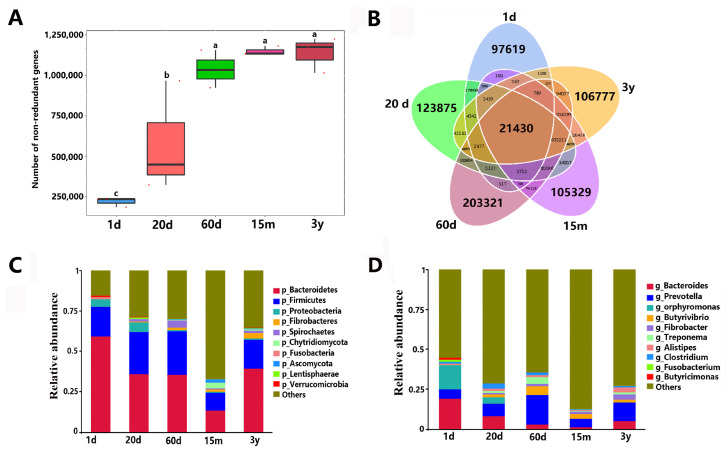
Gene prediction and relative abundance of rumen microbial in five developmental stages. (**A**) Box plot of gene number differences between groups. Different lowercase letters between groups indicated significant differences (*p* < 0.05), while the same lowercase letters indicated no significant differences (*p* > 0.05). (**B**) Venn diagram of gene numbers. (**C**) Histogram of relative abundance at phylum level. (**D**) Histogram of relative abundance at genus level.

This metagenome sequencing experiment generated 62,221.18 Mbp of raw data in total. After quality control, a total of 162,077.15 Mbp of clean data were obtained for all samples. A total of 4,042,703 ORFs and 2,734,302 non-redundant genes were predicted in all groups. In the five developmental stages, the number of non-redundant genes increased with age. the 60 d group, the 15 m group and the adult group showed no significant differences in the number of genes, but the number of genes in the three groups were significantly higher than those in the 1 d and 20 d groups, and the number of genes in the 20 d group was significantly higher than that in the 1 d group (Figure 5A). There were 21,430 common microbial genes across the five different age groups. There were 97,619, 123,875, 203,321, 105,329 and 10,677 unique genes in the 1 d, 20 d, 60 d, 15 m and adult groups, respectively (Figure 5B). 

Taxonomic classification was performed using DIAMOND software to map the sequence of non-redundant gene catalog against the NR database (e-value ≤ 1 × 10^−5^). There were 1,951,597 non-redundant genes among these sequences annotated by the NR database. The proportions of these genes annotated to the kingdom, phylum, class, order, family, genus and species levels were 76.71%, 71.14%, 66.81%, 66.19%, 57.39%, 53.48% and 34.12%, respectively. Metagenomics analysis also showed that the rumen of yaks from birth to adulthood was colonized by bacteria, eukaryota, archaea and viruses, while bacteria remained predominant (Supplemental Figure S4A, Supplemental Table S11). Based on the relative abundance of different classification levels, the top 10 relative abundances in each group were listed, and the rest were set to others (Figure 5C,D). We found that Bacteroidetes and Firmicutes were the dominant phylum in all groups, with 59.36%, 18.44% in the 1 d group, 35.94%, 26.23% in the 20 d group, 35.58%, 26.83% in the 60 d group, 13.33%, 11.06% in the 15 m group and 39.32%, 17.89% in the 3 y group, respectively (Supplemental Table S11). However, there were significant differences in microbial diversity and abundance at the five different stages. At the phylum level, the relative abundances of Bacteroidetes, Fusobacteria and Verrucomicrobia were higher in the 1 d group than those in the other groups. On the contrary, Fibrobacteres, Spirochaetes, Chytridiomycota, Ascomycota and Lentisphaerae were the lowest in the 1 d group. The relative abundance of Firmicutes was comparable in the 20 d and 60 d groups, but higher than those in other groups (Figure 5C). At the genus level, the relative abundances of *Bacteroides*, *Porphyromonas*, *Fusobacterium* and *Butyricimonas* were significantly higher in the 1 d group than those in the other groups. However, *Butyrivibrio*, *Fibrobacter* and *Treponema* in 1 d group were significantly lower than those in other groups (Figure 5D). Compared with the other groups, the dominant flora could be observed in different age groups. At the species level, compared with other groups, the dominant flora were *Fournierella massiliensis*, *Fusobacterium russii*, *Butyricimonas virosa*, *Bacteroides helcogenes*, *Desulfovibrio piger*, *Porphyromonas endodontalis*, *Porphyromonas gulae*, *Mycobacterium malmesburyense* and *Firmicutes bacterium CAG:555*, etc. in the 1 d group; *Porphyromonas levii*, *Bacteroides pyogenes*, *Clostridium sp. CAG:1024*, *Prevotella bryantii*, *Mycobacterium malmesburyense* and *Bacterium F083* in the 20 d group; *Butyrivibrio proteoclasticus*, *Selenomonas ruminantium*, *Treponema bryantii*, *Prevotella brevis*, *Prevotella ruminicola*, *Ruminococcus flavefaciens* and *Butyrivibrio fibrisolvens*, etc. in the 60 d group; *Butyrivibrio fibrisolvens* and *Neocallimastix californiae* in the 15 m group; *Bacterium P201*, *Succiniclasticum ruminis*, *Clostridiales bacterium*, *Bacteroidales bacterium WCE2008*, and *Bacteroidales bacterium WCE2004*, etc. in the 3 y group (Supplemental Figure S4D and Table S12). 

#### 3.3.2. CAZyme Functional Annotation

**Figure 6:**
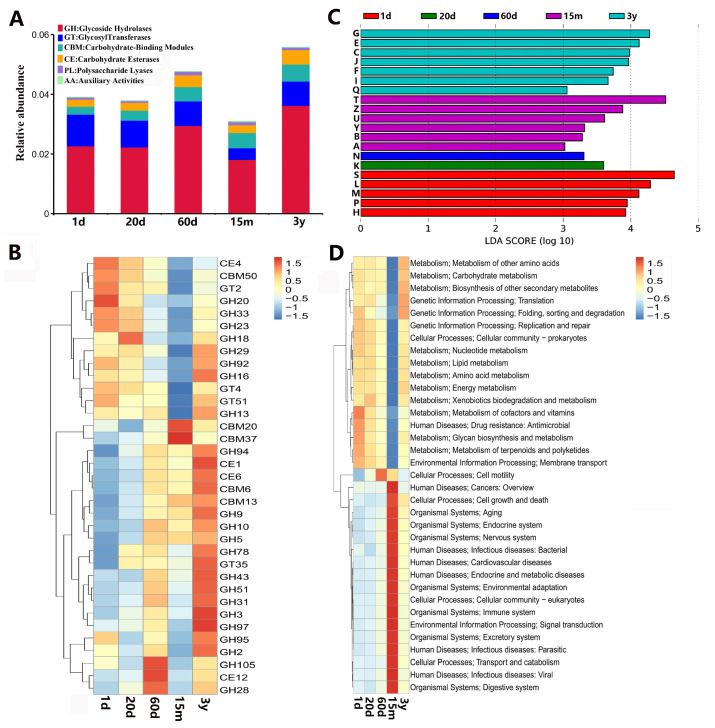
Functional difference of ruminal microbiome of yaks. (**A**) Relative abundance of CAZy enzymes at class level. (**B**) Heatmap of differences in CAZy enzymes at family level. (**C**) LEfSe analysis of eggNOG functional differences at level 1 (LDA > 3, *p* < 0.05). (**D**) Heatmap of top 35 abundant KEGG level 2 functional categories among 1 d, 20 d, 60 d, 15 m and 3 y based on z-standardized values of normalized relative abundance. (**B**,**D**) Horizontal axis represents sample groups, and vertical axis represents metabolic enzymes or pathways. The value corresponding to the heatmap is the Z value obtained after the standardization of the functional relative abundance of each row, that is, the Z value of a sample in a certain category is the difference between the relative abundance of the sample in the category and the average relative abundance of all samples in the category divided by the standard deviation of all samples in the category.

A total of 107,334 genes were annotated to the CAZy database, and the relative abundance in the 15 m group was the lowest. In five different developmental stages, glycoside hydrolase (GH) genes were the most highly represented, followed by glycosyltransferases (GTs), carbohydrate-binding modules (CBMs) and carbohydrate esterases (CEs), and a few genes were associated with polysaccharide lyases (PLs) and encoded auxiliary activities (AAs) (Figure 6A). Among them, the relative abundances of GHs, CEs and CBMs were the highest in the 3 y group, where the proportion of genes encoding CBMs increased with age. The relative abundance of GTs was the highest in the 1 d group and the lowest in the 15 m group. In addition, the relative abundance of PLs was the highest in the 60 d group and the lowest in the 20 d group. The expression of AAs was low in all groups, but was the highest in the 15 m group (Supplemental Figure S5A and Table S13). At the family level, GH20, GH23, GH33, GT2, GT4, GT51, CE4 and CBM50 were predominant in the 1 d group; GH18, GH23, GH33, CE4 and CBM50 were the main categories in the 20 d group; GH28, GH105 and CE12 were principal in the 60 d group; CBM20 and CBM37 were predominant in the 15 m group; and GH2, GH3, GH5, GH9, CE1, CE6, CBM6, CBM13 and GT35, etc. were dominant in the 3 y group (Figure 6B). At the family level, LDA effect size (LEfSe) analysis (LDA > 3, *p* < 0.05) showed that 27 CAZymes were enriched in the 1d group, including 15 GHs, 7 GTs, 3 CEs, 1 CBM, and 1 PL, while the GT2 was dominant; 13 differential CAZymes were enriched in 20 d group, including 7 GTs, 5 GHs and 1 CE, while the GT4 was principal; GH28, GH115, GH35 and CMB34 were enriched in the 60 d group; 36 differential CAZymes were involved in the 15 m group, including 17 CBMs, 10 GHs, 4 CEs, 3 PLs and 2 GTs, while the GH5 was the dominant; 10 differential CAZymes were enriched in the 3 y group, including 6 GHs, 2 CEs and 1 CBM, while the GH43 was principal (Supplemental Figure S5B).

#### 3.3.3. eggNOG Functional Annotation

Annotation of a cluster of orthologous groups of proteins for 1,358,731 nonredundant genes was performed using Diamond based on the eggNOG database (e-value ≤ 1 × 10^−5^). Most of these genes were ascribed to function unknown (S), carbohydrate transport and metabolism (G), amino acid transport and metabolism (E), replication, recombination and repair (L), cell wall/membrane/envelope biogenesis (M), translation, ribosomal structure and biogenesis (J), energy production and conversion (C), and inorganic ion transport and metabolism (P) in the eggNOG database (Supplemental Figure S6). LEfSe analysis revealed that the five pathways significantly enriched in the 1 d group were involved in function unknown (S), replication, recombination and repair (L), cell wall/membrane/envelope biogenesis (M), inorganic ion transport and metabolism (P), and coenzyme transport and metabolism (H); transcription (K) was significantly enriched in the 20 d group; cell motility (N) was markedly enriched in the 60 d group; signal transduction mechanisms (T), cytoskeleton (Z), intracellular trafficking, secretion and vesicular transport (U), nuclear structure (Y), chromatin structure and dynamics (B) and RNA processing and modification (A) were greatly enriched in the 15 m group; carbohydrate transport and metabolism (G), amino acid transport and metabolism (E), energy production and conversion (C), translation ribosomal structure and biogenesis (J), nucleotide transport and metabolism (F), lipid transport and metabolism (I) and secondary metabolites biosynthesitransport and catabolism (Q) were more abundant in the 3 y group (Figure 6C).

#### 3.3.4. KEGG Functional Annotation

The KEGG annotation was conducted using Diamond against the Kyoto Encyclopedia of Genes and Genomes database (e-value ≤ 1 × 10^−5^). A total of 1,384,438 nonredundant genes were annotated. In the first-level category, “Metabolism”, “Genetic information processing”, “Environment information processing” and “Cellular processes” were the dominant pathways (Supplemental Figure S7). Moreover, the heatmap of the top 35 KEGG functional categories at level 3 are presented in Figure 6D. Among the 35 categories, 10, 1, 1, 17 and 6 were notably enriched in the 1 d, 20 d, 60 d, 15 m and 3 y groups, respectively. In addition, the pathways enriched in the 15 m group were completely different from those in the other four groups, and mainly included those related to Human Diseases, Cellular Processes, Organismal Systems and Environmental Information Processing. However, in the other four groups, the primary pathways were related to Metabolism (metabolism of other amino acids, carbohydrate metabolism, biosynthesis of other secondary metabolites, nucleotide metabolism, lipid metabolism, amino acid metabolism, energy metabolism, etc.), Genetic Information Processing (translation, folding, sorting and degradation, and replication and repair), and Cellular Processes (cell motility). Among them, cell motility was significantly concentrated in the 60 d group, which was consistent with the eggNOG functional annotation.

#### 3.3.5. Metagenome–Host Transcriptome Interactions Influence Rumen Epithelial Development and Metabolism

**Figure 7:**
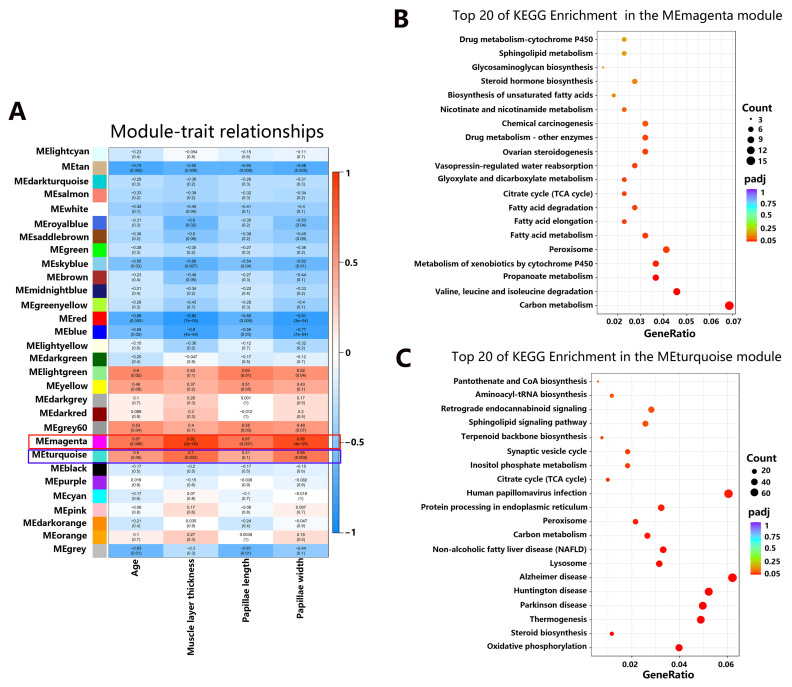
Heatmap of correlation between gene modules and phenotypic traits. WGCNA identification of rumen tissue gene modules correlated with the rumen and development indexes, and KEGG analysis of significant modules. (**A**) WGCNA of the correlation of host transcriptome with the rumen development indexes. (**B**) Top KEGG pathway of genes significantly in the MEmagenta module. (**C**) Top KEGG pathway of genes significantly in the MEturquoise module.

Host–microbial interactions in the developing rumen were evaluated by determining associations between rumen transcriptomes, age, muscle layer thickness, papillae length and width, and the microbial metagenomes. RNA-seq-based transcriptome profiling revealed a total of 17,494 genes expressed in the rumen tissue. The genes expressed in all rumen samples were grouped into 30 gene modules using WGCNA. These gene modules displayed various associations with these phenotypic traits (age, muscle layer thickness, papillae length and width). Among these modules, the expression of host genes in the MEred module and MEblue module was significantly negatively correlated, while the expression of genes in the MEmagenta and MEturquoise were positively correlated with these phenotypic traits (Figure 7A, Supplemental Table S14). The MEturquoise module enriched a total of 316 signaling pathways, of which 19 were significantly enriched, all of which were closely related to immune response and metabolism (Supplemental Table S14). These pathways are involved in oxidative phosphorylation, steroid biosynthesis, thermogenesis, lysosome, carbon metabolism and peroxisome, etc. (*q* value < 0.05; Figure 7C). In the MEmagenta module, a total of 250 signaling pathways were enriched, of which 14 were significantly enriched, almost all of which were related to metabolism (Supplemental Table S14). These pathways included carbon metabolism, valine, leucine and isoleucine degradation, propanoate metabolism, metabolism of xenobiotics by cytochrome P450, peroxisome, fatty acid metabolism and citrate cycle, etc. (*q* value < 0.05; Figure 7B).

**Figure 8:**
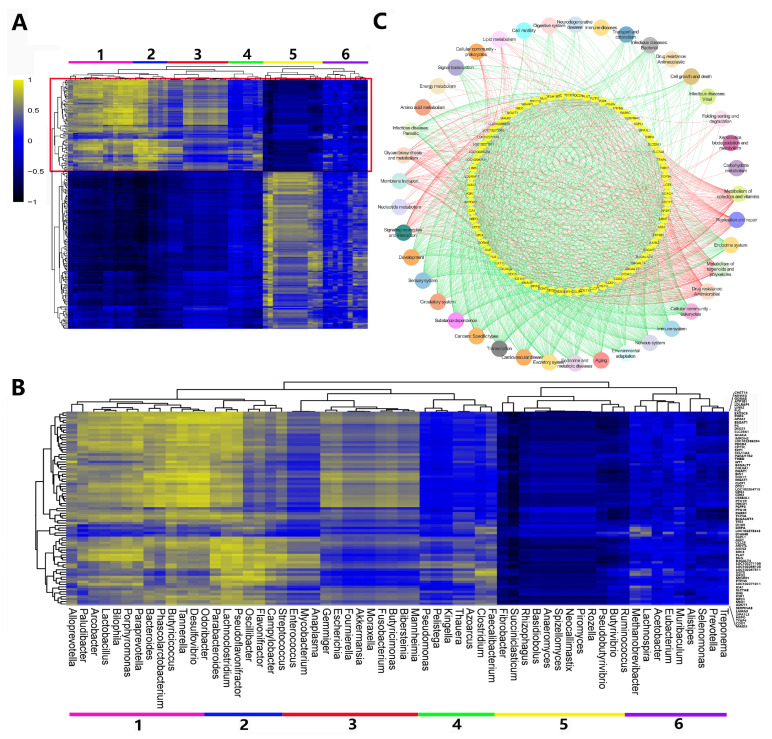
Associations among the transcriptome networks (gene modules), phenotypic traits and flora composition (taxonomy-genus level). (**A**) Association between the host genes co-expressed in the MEmagenta module and rumen content-associated bacterial genera relative abundance. (**B**) Bacterial clusters associated with ion binding-related genes co-expressed in the MEmagenta module. Cluster 1 (*Alloprevotella*, *Paludibacter*, *Arcobacter*, *Lactobacillus*, *Bilophila*, *Porphyromonas*, *Paraprevotella*, *Bacteroides*, *Phascolarctobacterium*, *Butyricicoccus*, *Tannerella*, *Desulfovibrio* and *Odoribacter*) positively correlates to the expression of the ion binding, regulation of cell cycle and transcription regulatory activity-related genes. Cluster 5 (*Ruminococcus*, *Butyrivibrio*, *Pseudobutyrivibrio*, *Rozella*, *Piromyces*, *Neocallimastix*, *Spizellomyces*, *Anaeromyces*, *Basidiobolus*, *Rhizophagus*, *Succiniclasticum* and *Fibrobacter*) negatively correlates to the expression of the ion binding, regulation of cell cycle and transcription regulatory activity-related genes. (**C**) Level 2 microbial functions associated with (*p* < 0.05, r > 0.6) host genes involved in rumen epithelial tissue development in the MEmagenta module, and red indicates positive correlation and green indicates negative correlation.

The MEmagenta module, which clustered host genes related to “rumen growth and metabolism” and positively correlated with age, muscle layer thickness, papillae length and width, was subjected to further analysis to explore the role of microflora in rumen development. Clustering of the correlation coefficient between the gene expression and the relative abundance of bacterial genera revealed six bacterial clusters depending on their association patterns (Figure 8A). A cluster (cluster 1) consisting of *Alloprevotella*, *Paludibacter*, *Arcobacter*, *Lactobacillus*, *Bilophila*, *Porphyromonas*, *Paraprevotella*, *Bacteroides*, *Phascolarctobacterium*, *Butyricicoccus*, *Tannerella*, *Desulfovibrio* and *Odoribacter* was positively correlated with the expression of 79 host genes involved in ion binding, regulation of cell cycle and transcription regulation (Figure 8B,C). The vast majority of “ion binding” host genes (22/79) were related to metal ion binding, including potassium, sodium, calcium, zinc, iron. Another cluster (cluster 5) containing genera mainly from Firmicutes, Eumycota and Fibrobacteres was negatively correlated with the expression of the same set of genes (Figure 8B). Among the level 2 microbial functions, metabolism, replication and repair, immune system, transcription, environmental adaptation, development, signaling molecules and interaction and membrane transport were strongly linked to the expression of host genes. Among these correlated host genes, there were 62 of 79 genes related to “rumen epithelium development” (*B3GAT1*, *CHST14*, *COL9A1*, *CREB3L1, DEGS1*, etc.), “rumen tissue metabolism” (*B4GALT7*, *FUS*, *GORAB*, *GPD2*, *H6PD, LIN52*, etc.), “rumen tissue immune system” (*AXIN2*, *ATP1B1*, *B3GAT1*, *AP2A1, CHST14*, etc.), and “membrane transport” (*ARNTL*, *EDC3*, *EXOSC8, GORAB*, etc.) (Figure 8C, Supplemental Table S15).

## 4. Discussion

Yaks are in a natural grazing state all the year round and take natural forage as the main source of nutrition [39]. Rumen epithelium is a unique place of interaction between host and microorganism that plays a key role in host nutrient metabolism, energy cycle and immune response [40,41]. Currently, there are few studies on the interaction between rumen microbiota and host genes in yaks from birth to adulthood. The present study integrated rumen epithelial morphology, epithelial transcriptomics and metagenomic analysis to explore the interaction between the rumen host and microbiota of yaks at different ages, thereby revealing the regulatory mechanism of yak adaptability to the plateau. Papillae length, papillae width and muscle layer thickness of rumen are the most important indexes to evaluate rumen morphology and development. The rumen morphological and physiological changes of yaks are closely related to age, growth environment, nutrition level and other factors [42,43,44]. In this study, rumen muscle layer thickness, papillae length and width of yaks increased with age. The morphological changes of slices showed that rumen muscle layer and papillae were well developed, and rumen muscle layer thickness increased significantly from 1 day to 3 years of age (*p* < 0.05), while the length and width of papillae increased significantly with age after 20 days (*p* < 0.05), suggesting that the development of the rumen muscle layer may be earlier than that of papillae in yaks. However, it was found that rumen papillae length, width and muscle layer thickness increased significantly after 28 days of age, but not after 21 days of age, although cashmere lambs began to eat concentrate and high quality alfalfa freely with ewes after 21 days of age [26], which is inconsistent with the results of this study. This may be due to the fact that yaks are naturally grazed and free foraging is not controlled, a certain degree of early feeding may effectively stimulate earlier rumen development, and a small amount of forage is found in the rumen of 20-day-old calves. Therefore, rumen muscle thickness and papillae development of yaks are earlier than those of cashmere goats, which may be caused by early free feeding of yak calves, or it may be caused by breed specificity. 

From the Venn diagrams of seven comparison groups, common DEmRNAs were not detected, and the unique genes observed were 112, 220, 603, 447, 47, 94 and 15, respectively. These results indicated that yaks at different ages had specific gene expressions, and the number of specific genes increased with age, but decreased at the age of 3 years, which may indicate that the yaks were mature and some development-related genes were not expressed. In the consecutive groups, more unique genes were found in the 20 vs. 1d (112) and 15 vs. 60 d (94) groups, while fewer unique genes were found in the 60 vs. 20 d (47) and 3 y vs. 15 m (15) groups. The lower number of unique genes indicated that the physiological states of the two groups were more similar. Twenty days old and 15 months old may be two important time nodes in the growth and development regulation of yaks. These results provide a theoretical basis for early weaning and sexual maturation of yaks. Additionally, the top five genes expressed in the seven comparison groups were analyzed. Among them, *S100A12*, *S100A9* and *LOC102283503* (lysozyme C) are closely related to immune regulation [45,46,47], *LOC102266213* (carbonic anhydrase 1) participates in the regulation of the acid–base balance in the stomach, while *A2ML1*, *KRT6A*, *DSP* and *LOC102268548* (keratin) are involved in cell growth, proliferation and differentiation [48,49,50,51]. *S100A12* and *S100A9* belong to a subfamily of the S100 family, called calgranin or myelin proteins, which are closely related to innate immune [45,46]. Lysozyme is an innate immune defense factor. Lysozyme secreted in ruminant stomachs has undergone much adaptive evolution in order to better adapt to the protease and acidic environment in the stomach, and exhibits the characteristics of acid resistance, heat resistance and pepsin resistance, which are different from conventional lysozyme, and can stably exist in the stomach environment and play the role of cracking the cell wall of rumen microorganisms [47]. Carbonic anhydrase belongs to the zinc metalase family, which can catalyze reversible hydration of carbon dioxide and maintain the acid–base balance in the stomach [48,49,50,51,52]. A2ML1, a member of the α-macroglobulin superfamily of proteins, encodes the secreted protease inhibitor α-2-macroglobulin-like-1, which is mainly involved in the defense mechanisms and maintenance of epidermal homeostasis [53]. The upregulation of *A2ML1* is associated with the differential expression of multiple genes, especially genes involved in keratinocyte and epidermal cell differentiation pathways [49]. KRT6A, a type II keratin protein, belongs to the keratin protein family which is a critical component of cytoskeleton in mammalian cells. It was found that KRT6A is involved in epidermatization of squamous epithelium and plays an important role in cell growth, proliferation and migration, especially keratinocyte migration [50,54,55]. In the present study, *KRT6A* and *LOC102283503* were observed in four closed populations, which may be due to the natural grazing of yaks from small to large, harsh natural environment, unstable food sources, mixed species, and rumen with good self-defense and self-growth repair functions, which are conducive to survival and health.

Previous studies have revealed that the rumen development process could be divided into three phases: non-rumination (0~3 weeks), transition (3~8 weeks), and rumination (8 weeks later) [27,56]. This was basically consistent with what we observed in the rumen of yaks, that is, there was no solid content in group 0 d, less forage and more milk in group 20 d, more forage and more milk in group 60 d, and only forage in group 15 m and group 3 y, indicating that yaks entered the transition stage of ruminant earlier. However, studies on rumen growth, the development and metabolism of yaks from non-ruminant to ruminant are far behind those of other ruminants [57,58]. To further explore the role of DEmRNAs in rumen functional transformation, KEGG and GO analyses were performed in four consecutive groups. KEGG pathway analysis showed that 40, 6, 8 and 5 signaling pathways were significantly upregulated, and 1, 0, 11 and 6 signaling pathways were significantly downregulated in four consecutive groups. Among them, the 40 dramatically upregulated signaling pathways at 20 d were mainly associated with immune response, metabolism and growth and development related pathways, which was consistent with the results of GO analysis. GO items were mainly concentrated in defense response to other organism, response to bacterium, polyketide metabolic process, glycoside metabolic process and mitotic nuclear division. In addition, the metabolic processes of polyketide and glycoside further indicated that the 20-day-old rumen was mainly dominated by glucose metabolism, and rumen microorganisms (bacteria, fungi, etc.) had colonized and started to work, and produced secondary metabolites-polyketides [59,60]. These results indicated that yaks can enter the transition stage earlier than other ruminants, so supplementary feeding of calves at 20 days of age or earlier can be considered in actual production. It was found that the growth and development rate of yak calves after weaning and supplementary feeding at 7 and 15 days after birth was better than that of traditional grazing yaks, which was basically consistent with the data of this study [61].

Rumen microbial community composition changed with the developmental stage [17,62]. However, the microbial population dynamics from newborn to adult yaks are poorly understood, as only a few studies have been reported on ruminal bacterial community in yaks from birth (5 days or 7 days after birth) to adulthood (2 years or 12 years) using 16S and 18S rRNA sequencing [19,63]. In the present study, metagenomics analysis was used to evaluate the composition and temporal dynamics of rumen microorganisms, from birth to adult, of Maiwa yak. We found that the number of non-redundant microbial genes increased with age, and there was no significant difference between the 60-day, 15-month, and 3-year groups. Metagenomics analysis also showed that the rumen of yaks from birth to adulthood was colonized by bacteria, eukaryota, archaea and viruses, but the proportions are obviously different, and bacteria remained predominant. Some studies have found that bacteria have been detected in the rumen of Israeli Holstein cows as early as birth (1 day old), and bacterial and fungal colonization have been observed in yak calves of 5 and 7 days of age, suggesting that bacterial colonization began long before rumen activity or even before ingestion of plant material [62]. In addition, archaea were detected at the age of 1 day, which was consistent with that of goats [41]. However, one study used 16S and 18S rRNA to sequence rumen microorganisms of yaks aged from 7 days to 12 years, and found that archaea were detected only after 14 days of age [19], which was inconsistent with this study, possibly due to different detection methods. We also found that the dominant phyla were Bacteroidetes and Firmicutes across all ages, which is in accordance with other recent studies on yaks [63]. In the 1 d and 20 d groups, the bacterial phylum Proteobacteria was significantly more numerous than that in other ages, which was consistent with previous studies that facultative anaerobic Proteobacteria are more adapted to the liquid-based dietary stage of early life [64,65]. The abundance of Fusobacteria and Verrucomicrobia were highest in the 1 d group and decreased sharply with age, which was consistent with that of the lambs [65,66]. Among them, there was a sudden increase in Verrucomicrobia in the 3-year-old group, but it was still lower than in the 1-day-old group. Previous studies have shown that Verrucomicrobia plays an important role in maintaining gut homeostasis and is mainly involved in the regulation of gastrointestinal immune function [67,68]. Notably, the abundance of genus *Akkermansia* and *Lactobacillus* in the 1 d group were significantly higher than that in all other four groups. *Akkermansia* is commonly found in the mucous layer of the gastrointestinal tract and is known for its mucin-degrading properties [69,70]. The latest study found that *Akkermansia* is almost not detected in fecal samples of captive yaks, while it is relatively high in free-range yaks at high altitude [71,72,73]. This indicates that the abundance of *Akkermansia* is not only related to age, but also closely related to the living environment. *Akkermansia* plays a crucial role in regulating nutrient requirements by mediating acetyl CoA from pyruvate into the TCA cycle, participating in the biosynthesis of arginine, and regulating the synthesis of fatty acids in the cold season with sparse forage [72]. In addition, *Akkermansia* can also maintain the body temperature of yaks at rest in extremely cold environments by promoting the absorption and metabolism of nutrients and generating heat through brown fat decomposition [71]. Studies have shown that *Lactobacillus* can regulate gastrointestinal immune function and promotes animal health. As a candidate for bacterial therapeutics, *Lactobacillus* has anti-pathogen effects, alleviating respiratory pathogens in cattle and preventing diseases in animals [73,74,75]. The abundances of the genera *Clostridium*, *Faecalibacterium*, *Methanobrevibacter* and *Pelistega* in the 20 d group were significantly higher than those in other groups. *Faecalibacterium* could produce butyrate, which has the function of anti-inflammatory, maintaining intestinal epithelium and balancing intestinal microbial community structure [76,77]. The abundance of Fibrobacteres increased with age and was highest at 3 years old. The Spirochaetes abundance was highest at 60 days of age, while the abundances of Chytridiomycota and Ascomycota at 15 months of age were significantly higher than those of other groups. These results suggested that each stage hosts its own distinct bacterial community and that the ruminal communities become more similar as age increases. According to the distribution characteristics of these bacteria, different types of feed and additives can be developed in practice to meet the rapid growth and development of yaks at different stages.

The species composition of rumen flora in yaks at different developmental stages was closely related to the types of rumen functional enzymes. It was found that 42 unique strains of cellulase- and GH-producing bacteria have been identified [78]. Functional analysis showed that 107,334 genes were annotated to the CAZy database. Except for the 15-month-old group, glycoside hydrolases (GHs) were predominant in all the other groups, but the types of GHs were different. CBM20 and CBM37 were dominant in the 15 m group. This may be closely related to the types of microorganisms in the rumen at different growth stages. In the present study, functional analysis revealed that the genera *Fournierella*, *Fusobacterium*, *Phascolarctobacterium*, *Moraxella*, *Butyricimonas*, *Akkermansia*, *Paraprevotella*, *Desulfovibrio* and *Porphyromonas* may contribute substantially to the production of GT2 and GH20; *Bacteroides*, *Lachnoclostridium*, *Streptococcus*, *Parabacteroides*, *Mycobacterium*, *Oscillibacter*, *Flavonifractor*, *Alloprevotella* and *Campylobacter* contribute to the production of CE4, CBM50, GT4, GT51, GH23, GH33, GH92, etc.; *Selenomonas*, *Butyrivibrio*, *Prevotella*, *Eubacterium*, *Ruminococcus* and *Treponema* contribute to the production of GH28, GH105 and CE12; *Piromyces* and *Neacallimastix* contribute to the production of CBM20 and CBM37; and *Alistipes*, *Fibrobacter* and *Succiniclasticum* contribute to CE1, CE6, GT35, CBM6, CBM13, GH43, GH51, etc. Previous studies showed that Bacteria of the Bacteroidetes phylum (*Butyricimonas*, *Paraprevotella*, *Bacteroides*, *Porphyromonas*, *Parabacteroides, Alloprevotella*, etc.) are considered primary degraders of polysaccharides, and can use thousands of enzyme combinations to break down glycans [79]. The Firmicutes (*Fournierella*, *Phascolarctobacterium*, *Lachnoclostridium, Streptococcus*, etc.) have been found to possess many genes responsible for fermenting dietary fiber, play a critical role in the degradation of complex plant carbohydrates, and can also interact with the intestinal mucosa, thereby contributing to gastrointestinal balance [80]. The genera *Piromyces* and *Neocallimastix* belong to the fungal phlum Chytridiomycota, and different fungi from Chytridomycetes have patterns of utilization of different carbon sources [81]. This suggests that Chytridomycetes may play important roles in carbohydrate metabolism. Moreover, Chytridiomycota CAZyme genes are relatively more abundant in cold environments such as the Southern Ocean than in tropical waters, and compared with the pelagic fungi basidiomycetes and ascomycetes, the pelagic Marine chytridobacteria do not produce and release secretory enzymes to the environment [82]. These results suggest that Chytridomycetes may be closely related to the plateau adaptation of yaks, and may adopt different ecological strategies.

Functional annotation based on the eggNOG database showed that the nonredundant genes in five different stage groups were mainly related to carbohydrate transport and metabolism, which was consistent with that of cattle yaks at different ages [57]. This can reflect the long-term grazing environment of yaks in the plateau, and yaks have the ability to resist roughage and stress in natural pastures. We found that transcription was more abundant in the 20 d group and cell motility was higher in the 60 d group when compared to the other four groups. It can be seen that there is a more active energy and material interaction between young yaks and their microbes. Cell motility is closely related to the integrating role of the plasma membrane [83]. The main characteristics of these aging cells are the function changes in cell membrane permeability, reduced metabolism, and substance transport function [84]. It was also found that the number of genes involved in energy production and transformation, carbohydrate transport and metabolism, amino acid transport and metabolism, and lipid transport and metabolism was higher in 3-year-olds as compared to the other four younger yaks groups. Thus, the adaptability of yaks to the plateau environment increases with age. For the KEGG functional analysis, a total of 1,384,438 nonredundant genes were annotated, and were mainly concentrated in metabolism and genetic information processing in the first-level category. Among the top 35 categories, 10, 1, 1, 17 and 6 were significantly enriched in the 1 d, 20 d, 60 d, 15 m and 3 y groups, respectively. In the 15 m group, these pathways were completely different from that in the other four groups, and mainly related to cell growth and death, endocrine system, signal transduction, the immune system, transport and catabolism and digestive system, cardiovascular diseases, endocrine and metabolic diseases, etc. However, these pathways in the other four groups mainly focus on nucleotide metabolism, lipid metabolism, amino acid metabolism, energy metabolism, carbohydrate metabolism, translation, folding, sorting and degradation, replication and repair, etc. For yaks, 15 months of age is an important stage of its transition from infancy to adulthood, and its growth and development pattern is naturally different from the other four stages. 

RNA-seq-based host transcriptome analysis has been extensively studied in ruminants to understand changes in rumen tissue with age, types of diet, weaning, and metabolic disorders at the molecular level [19,85,86,87]. The rumen separates the individual ruminant from the microbiome, but also interacts with the microbiome, thereby affecting the immune status and physiology of the host. The gut microbiome has the ability to reshape host chromatin, cause differential splicing, and directly interrupt the host signaling cascade [88]. This study investigated the changes of host transcriptome and microbiota and the molecular mechanisms behind host-microbial interactions during rumen development in yaks from birth to adulthood. The integrated analysis of host transcriptome and microbial metagenome further uncovered the underlying molecular mechanisms of rumen growth and development in yaks at five different developmental stages. Of the 30 host gene modules, only six (MEtan, MEskyblue, MEred, MEblue, MElightgreen and MEmagenta) were significantly negatively or positively associated with development of papillae, suggesting that only part of the host transcriptome was microbial-driven, while the rest was host-driven. Sequencing analysis of the gut microbiota and host in mice of different ages found that the gut microbiota dynamically modulates most of the epithelial transcriptome during postnatal development, but only targets a small number of microbially responsive genes through their DNA methylation status [89]. It has been also reported that about 10% of the intestinal transcriptome is regulated by microbiota in adult mice, mainly including genes with immune, cell proliferation and metabolic functions [90]. This is similar to the results of this study. We also found that two positively correlated modules, MEmagenta and MEturquoise, were significantly enriched with a large number of genes related to immune response, growth and metabolism. In the integrated analysis of host transcriptome and microbial metagenome in the MEmagenta module, we found six bacterial clusters depending on their association patterns. Among them, the expression of genes related to ion binding, cell cycle regulation and transcriptional regulation activity was positively correlated with cluster 1, and negatively correlated with cluster 5. We found that the majority of “ion binding” host genes were related to metal ion binding, including potassium, sodium, calcium, zinc and iron. This result is similar to the study in newborn Holstein bull calves, which also found that 49 host genes participated in ion binding, regulation of cell cycle, molecular functions, transcription regulatory activity and catalytic activity [22]. Metal ion salts have important effects on rumen growth and development in ruminants. The addition of the metal ions copper, cadmium and magnesium can affect the activity of some rumen enzymes which play an important role in nitrogen metabolism, and may further change the nitrogen metabolism in the rumen of sheep [91]. Zinc absorption also plays an vital role in early rumen papillae development and keratinization in goats [92]. Ion, metal ion, and calcium ion binding are also indispensable molecular activities occurring throughout follicle growth in yaks [93]. The present study revealed that 22 MEmagenta genes related to metal ion binding were correlated with the abundance of the bacterial genera (*Alloprevotella*, *Paludibacter*, *Arcobacter*, *Lactobacillus*, *Bilophila*, *Porphyromonas*, *Paraprevotella*, *Bacteroides*, *Phascolarctobacterium*, *Butyricicoccus*, *Tannerella*, *Desulfovibrio* and *Odoribacter*), suggesting that microbiota may influence rumen development through metal ions, including potassium, sodium, calcium, zinc and iron. The addition of these metal ions to yaks’ diets has been studied to understand its effects on milk production, hair and health [94,95]. However, their roles in rumen growth, development and metabolism, and the regulation of this process by microorganisms, are not well understood.

## 5. Conclusions

The present study found that yaks could enter the transition stage from pre-ruminant to ruminant earlier than other ruminants, so supplementary feeding of calves at 20 days of age or earlier can be considered in practical production. We also found that the yak rumen from birth to adulthood was colonized by bacteria, eukaryota, archaea and viruses, but the proportions are obviously different, and bacteria remained predominant. The integrated analysis of host transcriptome and microbial metagenome further found that only 6 out of 30 host gene modules were significantly negatively or positively associated with development of papillae, suggesting that only part of the host transcriptome is microbially driven. The present study also revealed that 22 MEmagenta genes related to metal ion binding were correlated with the abundance of the bacterial genera (*Alloprevotella*, *Paludibacter*, *Arcobacter*, *Lactobacillus*, etc.), suggesting that microbiota may influence rumen development through metal ions, including potassium, sodium, calcium, zinc and iron. This indicates that the microbiota may influence rumen development through metal ion absorption, and this interaction may be related to host mRNA regulation. It also provides a molecular basis for further research on weaning of calves at appropriate age and supplementary feeding of yaks at different stages, and also provides an important theoretical basis for research on yak nutrition and feeding management, so as to further improve economic benefits in the Qinghai-Tibet Plateau region. Therefore, these studies help us to understand the interaction between rumen host and microorganisms in yaks at different ages, and further provide a reliable theoretical basis for the development of feed and mineral element supplementation for yaks at different ages.

## Data Availability

All raw and processed sequencing data generated in this study have been submitted to the NCBI, metagenomic data with BioProject accession PRJNA1064652 (https://dataview.ncbi.nlm.nih.gov/object/PRJNA1064652?reviewer=40oftit3uln55srq5cgcj85fsg, accessed on 29 April 2024), and transcriptome data with the accession number GSE222396 (https://www.ncbi.nlm.nih.gov/geo/query/acc.cgi?acc=GSE222396, accessed on 29 April 2024).

## Supplementary Materials

The following supporting information can be downloaded at: https://www.mdpi.com/article/10.3390/ani14091365/s1, Table S1: Forward and reverse primers used for gene quantification by RT-qPCR; Table S2: The differentially expressed genes in the 20 d vs 1 d group; Table S3: The differentially expressed genes in the 60 d vs 1 d group; Table S4: The differentially expressed genes in the 15 m vs 1 d group; Table S5: The differentially expressed genes in the 3 y vs 1 d group; Table S6: The differentially expressed genes in the 60 d vs 20 d group; Table S7: The differentially expressed genes in the 15 m vs 60 d group; Table S8: The differentially expressed genes in the 3 y vs 15 m group; Table S9: The significantly enriched GO terms of differentially expressed genes in four consecutive groups; Table S10: The enriched KEGG pathways of differentially expressed genes in four consecutive groups; Table S11: The top 10 relative abundances at kingdom, phylum, class, order, family, genus and species levels; Table S12: The top 35 relative abundances at kingdom, phylum, class, order, family, genus and species levels; Table S13: The relative abundance of CAZy enzymes at level 1 in five developmental stages; Table S14: The KEGG enrichment in magenta, turquoise, red and tan modules of WGCNA; Table S15: Level 2 microbial functions associated with host genes in the MEmagenta module; Figure S1: The top 30 GO enrichment terms in four consecutive groups; Figure S2: The top 20 KEGG enrichment pathways in four consecutive groups; Figure S3: Transcription patterns of *BDH1*, *ECHS1*, *FDPS*, *HMGCS2* and *PCCA* compared to expression patterns in the RNA-seq in rumen; Figure S4: Heatmaps of the relative abundance at kingdom, phylum, genus and species levels; Figure S5. Heatmap and LEfSe analysis of CAZy enzymes; Figure S6: Distribution of eggNOG functional annotation of identified genes from rumen microbiota of yaks; Figure S7: The number of genes annotated in KEGG pathways at level 1.

## Abbreviations

DEGs	Differentially expressed genes
GHs	Glycoside hydrolases
GTs	Glycosyltransferases
CBMs	Carbohydrate-binding modules
CEs	Carbohydrate esterases
PLs	Polysaccharide lyases
AAs	Auxiliary activities
LEfSe	Linear discriminant analysis Effect Size
LDA	Linear Discriminant Analysis
eggNOG	Evolutionary genealogy of genes
FC	Fold change
KEGG	Kyoto Encyclopedia of Genes and Genomes
GO	Gene Ontolog
WGCNA	Weighted gene co-expression network analysis
RT-qPCR	Quantitative reverse transcription PCR
OD	Optical density
CAZy	Carbohydrate-active Enzymes
PL	Papillae length
PW	Papillae width
PCA	Principal component analysis

## References

[1] Ayalew W., Chu M., Liang C., Wu X., Yan P. (2021). Adaptation Mechanisms of Yak (*Bos grunniens*) to High-Altitude Environmental Stress. Animals.

[2] Jing X., Ding L., Zhou J., Huang X., Degen A., Long R. (2022). The adaptive strategies of yaks to live in the Asian highlands. Anim. Nutr..

[3] Long R., Dong S., Wei X., Pu X.P. (2005). The effect of supplementary feeds on the bodyweight of yaks in cold season. Livest. Prod. Sci..

[4] Xu T., Xu S., Hu L., Zhao N., Liu Z., Ma L., Liu H., Zhao X. (2017). Effect of dietary types on feed intakes, growth performance and economic benefit in Tibetan sheep and yaks on the Qinghai-Tibet Plateau during cold season. PLoS ONE.

[5] Meng G., La Y., Bao Q., Wu X., Ma X., Huang C., Chu M., Liang C., Yan P. (2023). Early Growth and Development and Nonlinear Model Fitting Analysis of Ashidan Yak. Animals.

[6] Baldwin R.L., Connor E.E. (2017). Rumen function and development. Vet. Clin. Food Anim. Pract..

[7] Diao Q., Zhang R., Fu T. (2019). Review of strategies to promote rumen development in calves. Animals.

[8] Kansagara Y., Savsani H., Chavda M., Chavda J., Belim S., Makwana K., Kansagara B. (2022). Rumen microbiota and nutrient metabolism: A review. Bhartiya Krishi Anusandhan Patrika.

[9] Negash A. (2022). Gut Microbiota Ecology Role in Animal Nutrition and Health Performance. J. Clin. Microbiol. Antimicrob..

[10] Zeineldin M., Barakat R., Elolimy A., Salem A.Z., Elghandour M.M., Monroy J.C. (2018). Synergetic action between the rumen microbiota and bovine health. Microb. Pathog..

[11] Reuben R.C., Elghandour M.M., Alqaisi O., Cone J.W., Márquez O., Salem A.Z. (2022). Influence of microbial probiotics on ruminant health and nutrition: Sources, mode of action and implications. J. Sci. Food Agric..

[12] Hess M., Sczyrba A., Egan R., Kim T.W., Chokhawala H., Schroth G., Luo S., Clark D.S., Chen F., Zhang T. (2011). Metagenomic discovery of biomass-degrading genes and genomes from cow rumen. Science.

[13] Liu K., Zhang Y., Yu Z., Xu Q., Zheng N., Zhao S., Huang G., Wang J. (2021). Ruminal microbiota–host interaction and its effect on nutrient metabolism. Anim. Nutr..

[14] Furman O., Shenhav L., Sasson G., Kokou F., Honig H., Jacoby S., Hertz T., Cordero O., Halperin E., Mizrahi I. (2020). Stochasticity constrained by deterministic effects of diet and age drive rumen microbiome assembly dynamics. Nat. Commun..

[15] Guo C.Y., Ji S.K., Yan H., Wang Y.J., Liu J.J., Cao Z.J., Yang H.J., Zhang W.J., Li S.L. (2020). Dynamic change of the gastrointestinal bacterial ecology in cows from birth to adulthood. MicrobiologyOpen.

[16] Zhang Y., Cai W., Li Q., Wang Y., Wang Z., Zhang Q., Xu L., Xu L., Hu X., Zhu B. (2022). Transcriptome analysis of bovine rumen tissue in three developmental stages. Front. Genet..

[17] Li L.P., Peng K.L., Xue M.Y., Zhu S.L., Liu J.X., Sun H.Z. (2022). An Age Effect of Rumen Microbiome in Dairy Buffaloes Revealed by Metagenomics. Microorganisms.

[18] Li B., Zhang K., Li C., Wang X., Chen Y., Yang Y. (2019). Characterization and Comparison of Microbiota in the Gastrointestinal Tracts of the Goat (*Capra hircus*) During Preweaning Development. Front. Microbiol..

[19] Guo W., Zhou M., Ma T., Bi S., Wang W., Zhang Y., Huang X., Guan L.L., Long R. (2020). Survey of rumen microbiota of domestic grazing yak during different growth stages revealed novel maturation patterns of four key microbial groups and their dynamic interactions. Anim. Microbiome.

[20] Zhang G., Wang Y., Luo H., Qiu W., Zhang H., Hu L., Wang Y., Dong G., Guo G. (2019). The Association between Inflammaging and Age-Related Changes in the Ruminal and Fecal Microbiota Among Lactating Holstein Cows. Front. Microbiol..

[21] Liu C., Meng Q., Chen Y., Xu M., Shen M., Gao R., Gan S. (2017). Role of Age-Related Shifts in Rumen Bacteria and Methanogens in Methane Production in Cattle. Front. Microbiol..

[22] Malmuthuge N., Liang G., Guan L.L. (2019). Regulation of rumen development in neonatal ruminants through microbial metagenomes and host transcriptomes. Genome Biol..

[23] Liu X., Sha Y., Lv W., Cao G., Guo X., Pu X., Wang J., Li S., Hu J., Luo Y. (2022). Multi-Omics Reveals That the Rumen Transcriptome, Microbiome, and Its Metabolome Co-regulate Cold Season Adaptability of Tibetan Sheep. Front. Microbiol..

[24] Hu R., Zou H., Wang Z., Cao B., Peng Q., Jing X., Wang Y., Shao Y., Pei Z., Zhang X. (2019). Nutritional Interventions Improved Rumen Functions and Promoted Compensatory Growth of Growth-Retarded Yaks as Revealed by Integrated Transcripts and Microbiome Analyses. Front. Microbiol..

[25] Huang C., Ge F., Yao X., Guo X., Bao P., Ma X., Wu X., Chu M., Yan P., Liang C. (2021). Microbiome and Metabolomics Reveal the Effects of Different Feeding Systems on the Growth and Ruminal Development of Yaks. Front. Microbiol..

[26] Pan X., Li Z., Li B., Zhao C., Wang Y., Chen Y., Jiang Y. (2021). Dynamics of rumen gene expression, microbiome colonization, and their interplay in goats. BMC Genom..

[27] Li K., Shi B., Na R. (2023). The Colonization of Rumen Microbiota and Intervention in Pre-Weaned Ruminants. Animals.

[28] Kim D., Langmead B., Salzberg S.L. (2015). HISAT: A fast spliced aligner with low memory requirements. Nat. Methods.

[29] Roberts A., Trapnell C., Donaghey J., Rinn J.L., Pachter L. (2011). Improving RNA-Seq expression estimates by correcting for fragment bias. Genome Biol..

[30] Trapnell C., Williams B.A., Pertea G., Mortazavi A., Kwan G., van Baren M.J., Salzberg S.L., Wold B.J., Pachter L. (2010). Transcript assembly and quantification by RNA-Seq reveals unannotated transcripts and isoform switching during cell differentiation. Nat. Biotechnol..

[31] Anders S., Pyl P.T., Huber W. (2015). HTSeq—A Python framework to work with high-throughput sequencing data. Bioinformatics.

[32] Anders S., Huber W. (2012). Differential Expression of RNA-Seq Data at the Gene Level—The DESeq Package.

[33] Young M.D., Wakefield M.J., Smyth G.K., Oshlack A. (2010). Gene ontology analysis for RNA-seq: Accounting for selection bias. Genome Biol..

[34] Kanehisa M., Araki M., Goto S., Hattori M., Hirakawa M., Itoh M., Katayama T., Kawashima S., Okuda S., Tokimatsu T. (2008). KEGG for linking genomes to life and the environment. Nucleic. Acids. Res..

[35] Langfelder P., Horvath S. (2008). WGCNA: An R package for weighted correlation network analysis. BMC Bioinform..

[36] Li W., Godzik A. (2006). Cd-hit: A fast program for clustering and comparing large sets of protein or nucleotide sequences. Bioinformatics.

[37] Fu L., Niu B., Zhu Z., Wu S., Li W. (2012). CD-HIT: Accelerated for clustering the next-generation sequencing data. Bioinformatics.

[38] Buchfink B., Xie C., Huson D.H. (2015). Fast and sensitive protein alignment using DIAMOND. Nat. Methods.

[39] Shi F., Guo N., Degen A., Niu J., Wei H., Jing X., Ding L., Shang Z., Long R. (2020). Effects of level of feed intake and season on digestibility of dietary components, efficiency of microbial protein synthesis, rumen fermentation and ruminal microbiota in yaks. Anim. Feed Sci. Technol..

[40] Lin L., Xie F., Sun D., Liu J., Zhu W., Mao S. (2019). Ruminal microbiome-host crosstalk stimulates the development of the ruminal epithelium in a lamb model. Microbiome.

[41] Petri R., Kleefisch M., Metzler-Zebeli B., Zebeli Q., Klevenhusen F.J.A., Microbiology E. (2018). Changes in the rumen epithelial microbiota of cattle and host gene expression in response to alterations in dietary carbohydrate composition. Appl. Environ. Microb..

[42] Jiao J., Li X., Beauchemin K.A., Tan Z., Tang S., Zhou C. (2015). Rumen development process in goats as affected by supplemental feeding v. grazing: Age-related anatomic development, functional achievement and microbial colonisation. Brit. J. Nutr..

[43] Wang B., Wang D., Wu X., Cai J., Liu M., Huang X., Wu J., Liu J., Guan L. (2017). Effects of dietary physical or nutritional factors on morphology of rumen papillae and transcriptome changes in lactating dairy cows based on three different forage-based diets. BMC Genom..

[44] Gupta M., Khan N., Rastogi A., Varun T. (2016). Nutritional drivers of rumen development: A review. Agric. Rev..

[45] Realegeno S., Kelly-Scumpia K.M., Dang A.T., Lu J., Teles R., Liu P.T., Schenk M., Lee E.Y., Schmidt N.W., Wong G.C. (2016). S100A12 Is Part of the Antimicrobial Network against Mycobacterium leprae in Human Macrophages. PLoS Pathog..

[46] Srikrishna G. (2012). S100A8 and S100A9: New insights into their roles in malignancy. J. Innate. Immun..

[47] Ferraboschi P., Ciceri S., Grisenti P. (2021). Applications of Lysozyme, an Innate Immune Defense Factor, as an Alternative Antibiotic. Antibiotics.

[48] Kivelä A.J., Kivelä J., Saarnio J., Parkkila S. (2005). Carbonic anhydrases in normal gastrointestinal tract and gastrointestinal tumours. World J. Gastroenterol..

[49] Larson E.D., Magno J.P.M., Steritz M.J., Llanes E., Cardwell J., Pedro M., Roberts T.B., Einarsdottir E., Rosanes R.A.Q., Greenlee C. (2019). A2ML1 and otitis media: Novel variants, differential expression, and relevant pathways. Hum. Mutat..

[50] Che D., Wang M., Sun J., Li B., Xu T., Lu Y., Pan H., Lu Z., Gu X. (2021). KRT6A Promotes Lung Cancer Cell Growth and Invasion Through MYC-Regulated Pentose Phosphate Pathway. Front. Cell Dev. Biol..

[51] Hao Y., Bates S., Mou H., Yun J.H., Pham B., Liu J., Qiu W., Guo F., Morrow J.D., Hersh C.P. (2020). Genome-Wide Association Study: Functional Variant rs2076295 Regulates Desmoplakin Expression in Airway Epithelial Cells. Am. J. Resp. Crit. Care..

[52] Occhipinti R., Boron W.F. (2019). Role of Carbonic Anhydrases and Inhibitors in Acid-Base Physiology: Insights from Mathematical Modeling. Int. J. Mol. Sci..

[53] Aoki Y., Niihori T., Inoue S., Matsubara Y. (2016). Recent advances in RASopathies. J. Hum. Genet..

[54] Yang B., Zhang W., Zhang M., Wang X., Peng S., Zhang R. (2020). KRT6A Promotes EMT and Cancer Stem Cell Transformation in Lung Adenocarcinoma. Technol. Cancer. Res. Treat..

[55] Wang F., Chen S., Liu H.B., Parent C.A., Coulombe P.A. (2018). Keratin 6 regulates collective keratinocyte migration by altering cell-cell and cell-matrix adhesion. J. Cell. Biol..

[56] Wang Z., Liang Y., Lu J., Wei Z., Bao Y., Yao X., Fan Y., Wang F., Wang D., Zhang Y. (2023). Dietary spirulina supplementation modifies rumen development, fermentation and bacteria composition in Hu sheep when consuming high-fat dietary. Front. Vet. Sci..

[57] Sha Y., Hu J., Shi B., Dingkao R., Wang J., Li S., Zhang W., Luo Y., Liu X. (2020). Characteristics and Functions of the Rumen Microbial Community of Cattle-Yak at Different Ages. Biomed. Res. Int..

[58] Ma L., Xu S., Liu H., Xu T., Hu L., Zhao N., Han X., Zhang X. (2019). Yak rumen microbial diversity at different forage growth stages of an alpine meadow on the Qinghai-Tibet Plateau. PeerJ.

[59] Liu C., Kakeya H. (2020). Cryptic Chemical Communication: Secondary Metabolic Responses Revealed by Microbial Co-culture. Chem. Asian. J..

[60] Nazzaro F., Fratianni F., d’Acierno A., De Feo V., Ayala-Zavala F.J., Gomes-Cruz A., Granato D., Coppola R. (2019). Effect of polyphenols on microbial cell-cell communications. Quorum Sensing.

[61] Wei J., Bai Q., Luo X., Guan J., An T., Zhao H., Tan W., Li H.d., Xie R., Sa Q. (2022). Effects of different weaning patterns on growth and development, serum biochemical indices and antioxidant capacity of calf yaks. Chin. Anim. Husb. Vet. Med..

[62] Jami E., Israel A., Kotser A., Mizrahi I. (2013). Exploring the bovine rumen bacterial community from birth to adulthood. ISME J..

[63] Yang S., Zhang G., Yuan Z., He S., Wang R., Zheng J., Mao H., Chai J., Wu D. (2023). Exploring the temporal dynamics of rumen bacterial and fungal communities in yaks (*Bos grunniens*) from 5 days after birth to adulthood by full-length 16S and 18S rRNA sequencing. Front. Vet. Sci..

[64] Guzman C.E., Bereza-Malcolm L.T., De Groef B., Franks A.E. (2015). Presence of Selected Methanogens, Fibrolytic Bacteria, and Proteobacteria in the Gastrointestinal Tract of Neonatal Dairy Calves from Birth to 72 Hours. PLoS ONE.

[65] Palma-Hidalgo J.M., Yáñez-Ruiz D.R., Jiménez E., Martín-García A.I., Belanche A. (2021). Presence of Adult Companion Goats Favors the Rumen Microbial and Functional Development in Artificially Reared Kids. Front. Vet. Sci..

[66] Yin X., Ji S., Duan C., Tian P., Ju S., Yan H., Zhang Y., Liu Y. (2021). Age-Related Changes in the Ruminal Microbiota and Their Relationship with Rumen Fermentation in Lambs. Front. Microbiol..

[67] Derrien M., Van Baarlen P., Hooiveld G., Norin E., Müller M., de Vos W.M. (2011). Modulation of Mucosal Immune Response, Tolerance, and Proliferation in Mice Colonized by the Mucin-Degrader *Akkermansia muciniphila*. Front. Microbiol..

[68] Shen H., Lu Z., Chen Z., Wu Y., Shen Z. (2016). Rapid Fermentable Substance Modulates Interactions between Ruminal Commensals and Toll-Like Receptors in Promotion of Immune Tolerance of Goat Rumen. Front. Microbiol..

[69] Sonnenburg E.D., Sonnenburg J.L. (2019). The ancestral and industrialized gut microbiota and implications for human health. Nat. Rev. Microbiol..

[70] Smits S.A., Leach J., Sonnenburg E.D., Gonzalez C.G., Lichtman J.S., Reid G., Knight R., Manjurano A., Changalucha J., Elias J.E. (2017). Seasonal cycling in the gut microbiome of the Hadza hunter-gatherers of Tanzania. Science.

[71] Wen Y., Li S., Wang Z., Feng H., Yao X., Liu M., Chang J., Ding X., Zhao H., Ma W. (2022). Intestinal Microbial Diversity of Free-Range and Captive Yak in Qinghai Province. Microorganisms.

[72] Guo N., Wu Q., Shi F., Niu J., Zhang T., Degen A.A., Fang Q., Ding L., Shang Z., Zhang Z. (2021). Seasonal dynamics of diet-gut microbiota interaction in adaptation of yaks to life at high altitude. npj Biofilms Microbi..

[73] Amat S., Alexander T.W., Holman D.B., Schwinghamer T., Timsit E. (2020). Intranasal Bacterial Therapeutics Reduce Colonization by the Respiratory Pathogen *Mannheimia haemolytica* in Dairy Calves. mSystems.

[74] Adetoye A., Pinloche E., Adeniyi B.A., Ayeni F.A. (2018). Characterization and anti-salmonella activities of lactic acid bacteria isolated from cattle faeces. BMC Microbiol..

[75] Fernández S., Fraga M., Silveyra E., Trombert A.N., Rabaza A., Pla M., Zunino P. (2018). Probiotic properties of native Lactobacillus spp. strains for dairy calves. Benef. Microbes.

[76] Sokol H., Pigneur B., Watterlot L., Lakhdari O., Bermúdez-Humarán L.G., Gratadoux J.J., Blugeon S., Bridonneau C., Furet J.P., Corthier G. (2008). *Faecalibacterium prausnitzii* is an anti-inflammatory commensal bacterium identified by gut microbiota analysis of Crohn disease patients. Proc. Natl. Acad. Sci. USA.

[77] Wrzosek L., Miquel S., Noordine M.L., Bouet S., Joncquel Chevalier-Curt M., Robert V., Philippe C., Bridonneau C., Cherbuy C., Robbe-Masselot C. (2013). *Bacteroides thetaiotaomicron* and *Faecalibacterium prausnitzii* influence the production of mucus glycans and the development of goblet cells in the colonic epithelium of a gnotobiotic model rodent. BMC Biol..

[78] Bashir Z., Kondapalli V.K., Adlakha N., Sharma A., Bhatnagar R.K., Chandel G., Yazdani S.S. (2013). Diversity and functional significance of cellulolytic microbes living in termite, pill-bug and stem-borer guts. Sci. Rep..

[79] Lapébie P., Lombard V., Drula E., Terrapon N., Henrissat B. (2019). Bacteroidetes use thousands of enzyme combinations to break down glycans. Nat. Commun..

[80] Sun Y., Zhang S., Nie Q., He H., Tan H., Geng F., Ji H., Hu J., Nie S. (2023). Gut firmicutes: Relationship with dietary fiber and role in host homeostasis. Crit. Rev. Food. Sci. Nutr..

[81] Gleason F.H., Marano A.V., Digby A.L., Al-Shugairan N., Lilje O., Steciow M.M., Barrera M.D., Inaba S., Nakagiri A. (2011). Patterns of utilization of different carbon sources by Chytridiomycota. Hydrobiologia.

[82] Baltar F., Zhao Z., Herndl G.J. (2021). Potential and expression of carbohydrate utilization by marine fungi in the global ocean. Microbiome.

[83] Keren K. (2011). Cell motility: The integrating role of the plasma membrane. Eur. Biophys. J..

[84] McHugh D., Gil J. (2018). Senescence and aging: Causes, consequences, and therapeutic avenues. J. Cell. Biol..

[85] Li W., Gelsinger S., Edwards A., Riehle C., Koch D. (2019). Transcriptome analysis of rumen epithelium and meta-transcriptome analysis of rumen epimural microbial community in young calves with feed induced acidosis. Sci. Rep..

[86] Wang J., Fan H., Li M., Zhao K., Xia S., Chen Y., Shao J., Tang T., Bai X., Liu Z. (2023). Integration of Non-Coding RNA and mRNA Profiles Reveals the Mechanisms of Rumen Development Induced by Different Types of Diet in Calves. Genes.

[87] Nishihara K., Kato D., Suzuki Y., Kim D., Nakano M., Yajima Y., Haga S., Nakano M., Ishizaki H., Kawahara-Miki R. (2018). Comparative transcriptome analysis of rumen papillae in suckling and weaned Japanese Black calves using RNA sequencing. J. Anim. Sci..

[88] Nichols R.G., Davenport E.R. (2021). The relationship between the gut microbiome and host gene expression: A review. Hum. Genet..

[89] Pan W.H., Sommer F., Falk-Paulsen M., Ulas T., Best P., Fazio A., Kachroo P., Luzius A., Jentzsch M., Rehman A. (2018). Exposure to the gut microbiota drives distinct methylome and transcriptome changes in intestinal epithelial cells during postnatal development. Genome Med..

[90] Sommer F., Nookaew I., Sommer N., Fogelstrand P., Bäckhed F. (2015). Site-specific programming of the host epithelial transcriptome by the gut microbiota. Genome Biol..

[91] Faixová Z., Faix Š. (2002). Influence of metal ions on ruminal enzyme activities. Acta. Vet. Brno.

[92] Černík J., Pavlata L., Pechová A., Mišurová Ľ., Jokverová O., Luňáček J., Halouzka R. (2014). Effects of peroral supplementation of different forms of zinc on the ruminal mucosa of goat kids—A morphometric study. Acta. Vet. Brno..

[93] Guo S., Cao M., Wang X., Xiong L., Wu X., Bao P., Chu M., Liang C., Yan P., Pei J. (2021). Changes in Transcriptomic Profiles in Different Reproductive Periods in Yaks. Biology.

[94] Li W., Li G., Jing J., Tian L., Bai X., Lu X., Cui Z., Li B. (2022). Research progress of trace element nutrition in yak. Heilongjiang Anim. Husb. Vet. Med..

[95] Zhao Z., Ma Z., Wang H., Zhang C. (2022). Effects of trace minerals supply from rumen sustained release boluses on milk yields and components, rumen fermentation and the rumen bacteria in lactating yaks (*Bos grunniens*). J. Anim. Feed. Sci. Technol..

