# Supplementary material for: Microbial Metagenomes and Host Transcriptomes Reveal the Dynamic Changes of Rumen Gene Expression, Microbial Colonization and Co-Regulation of Mineral Element Metabolism in Yaks from Birth to Adulthood
Source: Animals (Basel). 2024 Apr 30;14(9):1365. doi: 10.3390/ani14091365 (PMC11083404; doi:10.3390/ani14091365)
Supplement: Supplementary file 1 [file animals-14-01365-s001.zip › Table S1-Forward and reverse primers used for gene quantification by RT-qPCR.pdf]

Table S1. Forward and reverse primers used for gene quantifification by RT-qPCR

Gene	GenBank	Primer sequence(5'-3')	Amplicon Size (bp)
BDH1	XM_005897835.2	F: CTTCAGTGGACCCGGTTCG	112
		R: CCAGCAAACACAAGGAAGCC	
ECHS1	XM_005891447.2	F: AACTTTTGCCACCGAAGACC	135
		R: GCTTTTGAAGCTGCTCGTCC	
FDPS	XM_005898693.2	F: TACTAGATCCAGCGACCCGA	105
		R: AGGGAGTAAAATGCTCTGTTCCT	
HMGCS2	XM_005895274.2	F: GCAACACTGACATTGAGGGC	118
		R: ACCAGTGCATAGCGACCATC	
PCCA	XM_005899255.2	F: ACCATGAAAGTTCAAGAGTGCC	142
		R: TGTAGCAGCTTCTGTGGTTCA	
