# Supplementary figures and images for: Microbial Metagenomes and Host Transcriptomes Reveal the Dynamic Changes of Rumen Gene Expression, Microbial Colonization and Co-Regulation of Mineral Element Metabolism in Yaks from Birth to Adulthood
Source: Animals (Basel). 2024 Apr 30;14(9):1365. doi: 10.3390/ani14091365 (PMC11083404; doi:10.3390/ani14091365)

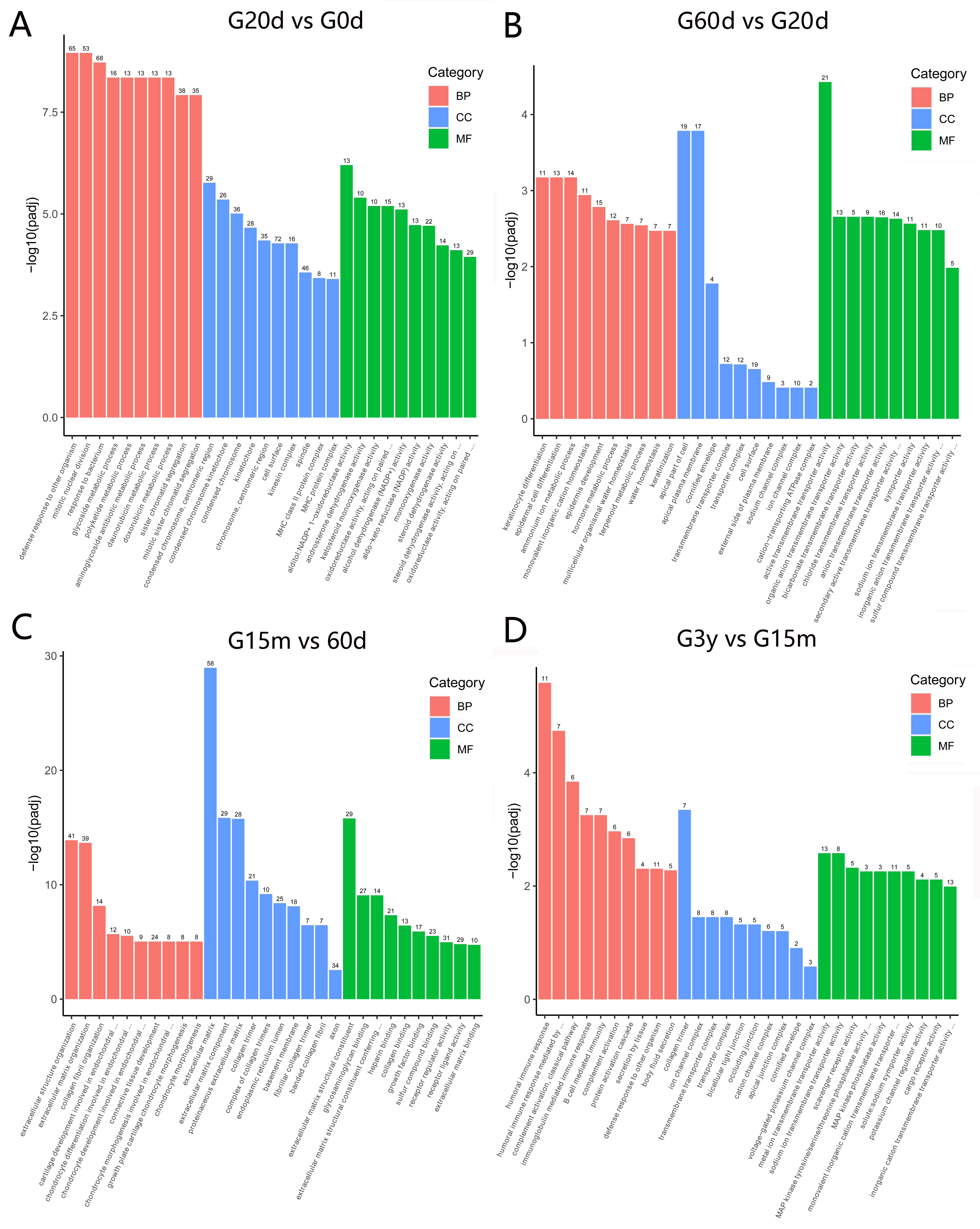

Supplement: Supplementary file 1 [file animals-14-01365-s001.zip › Figure S1-The top 30 GO enrichment terms in four consecutive groups.jpg]

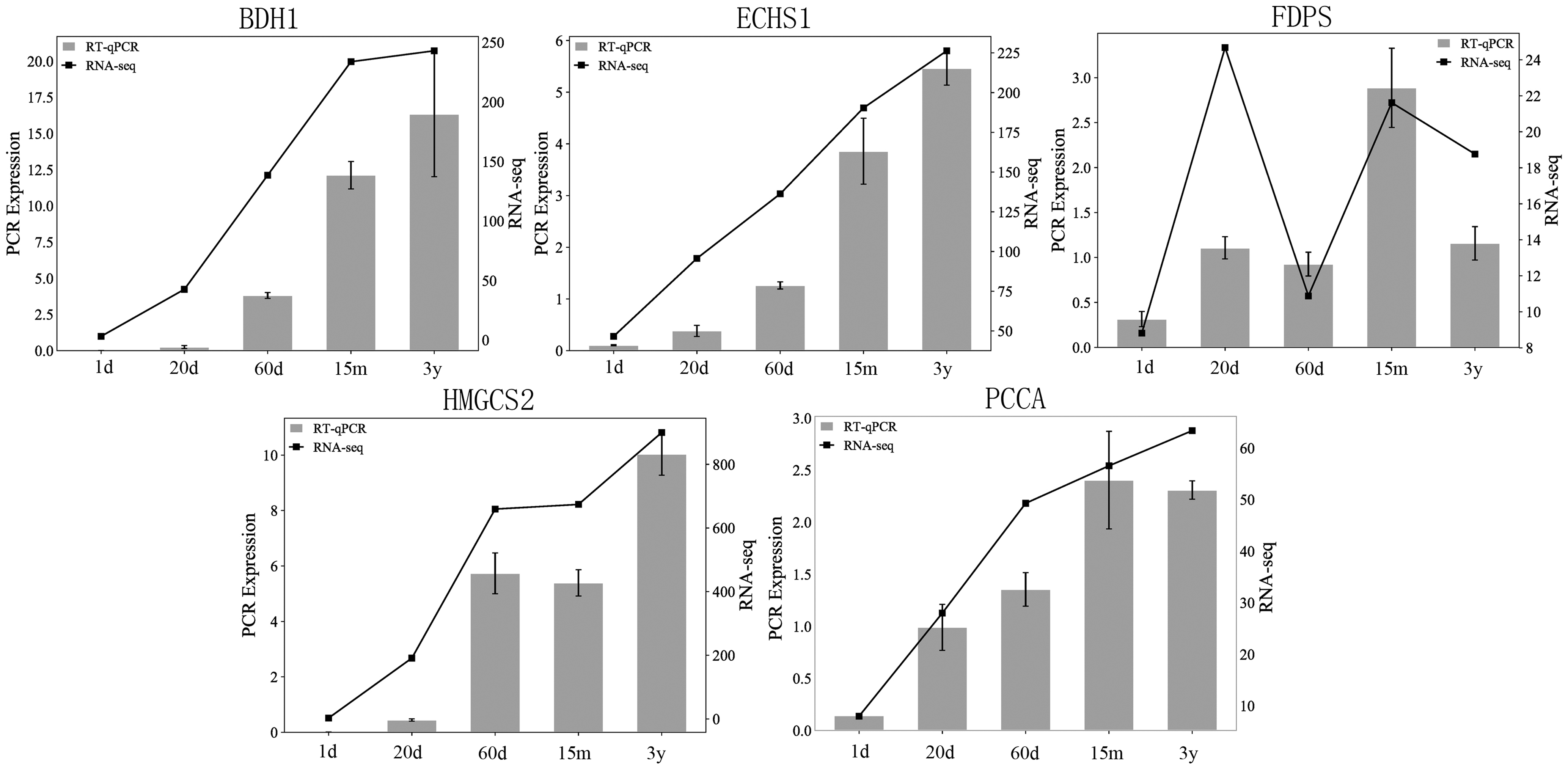

Supplement: Supplementary file 1 [file animals-14-01365-s001.zip › Figure S3-Transcription patterns of BDH1ú1⁄4ECHS1ú1⁄4FDPSú1⁄4HMGCS2 and PCCA compared to expression patterns in the RNA-seq in rumen.jpg]

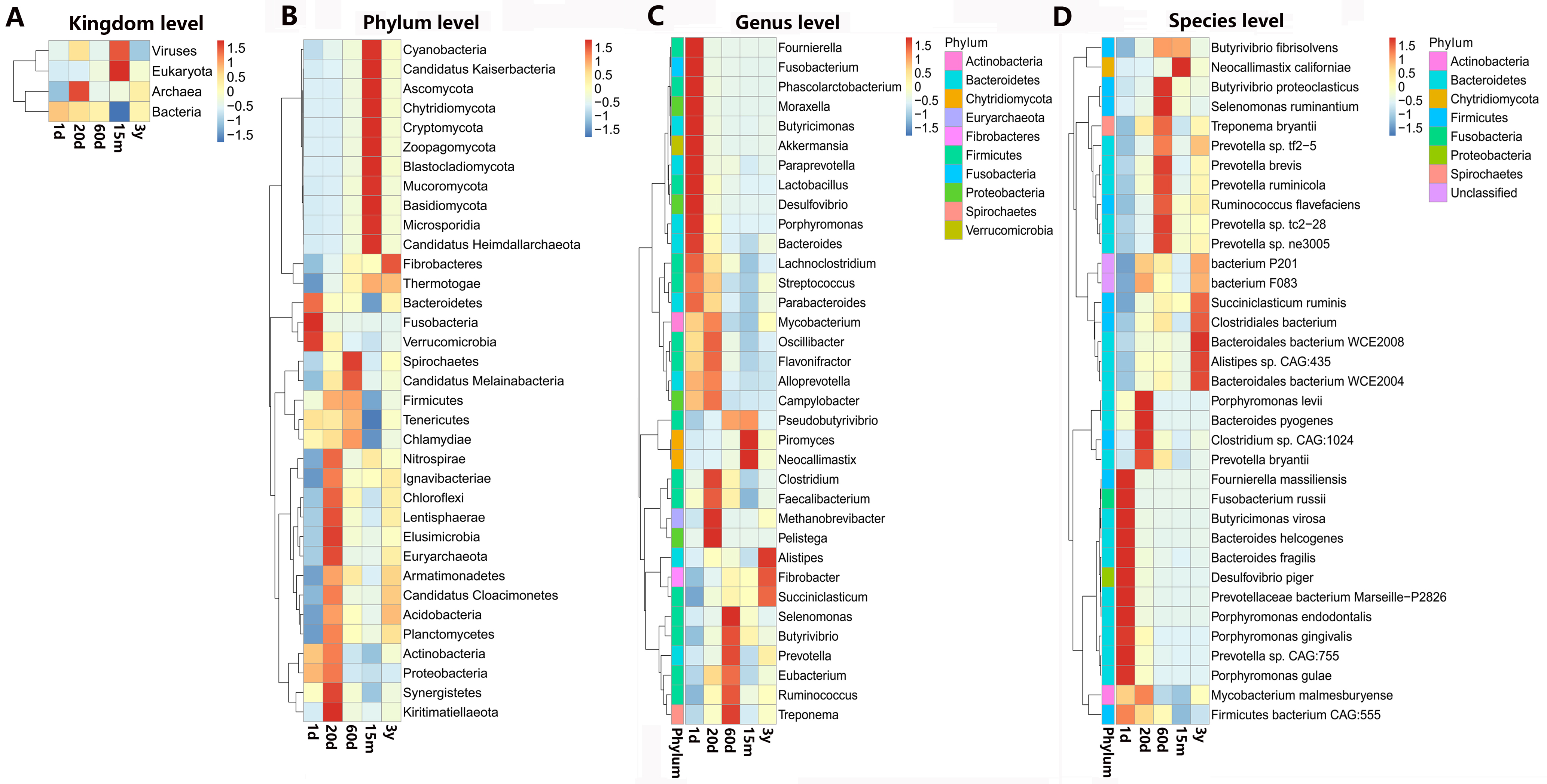

Supplement: Supplementary file 1 [file animals-14-01365-s001.zip › Figure S4-Heatmaps of the relative abundance at kingdom, phylum, genus and species levels.jpg]

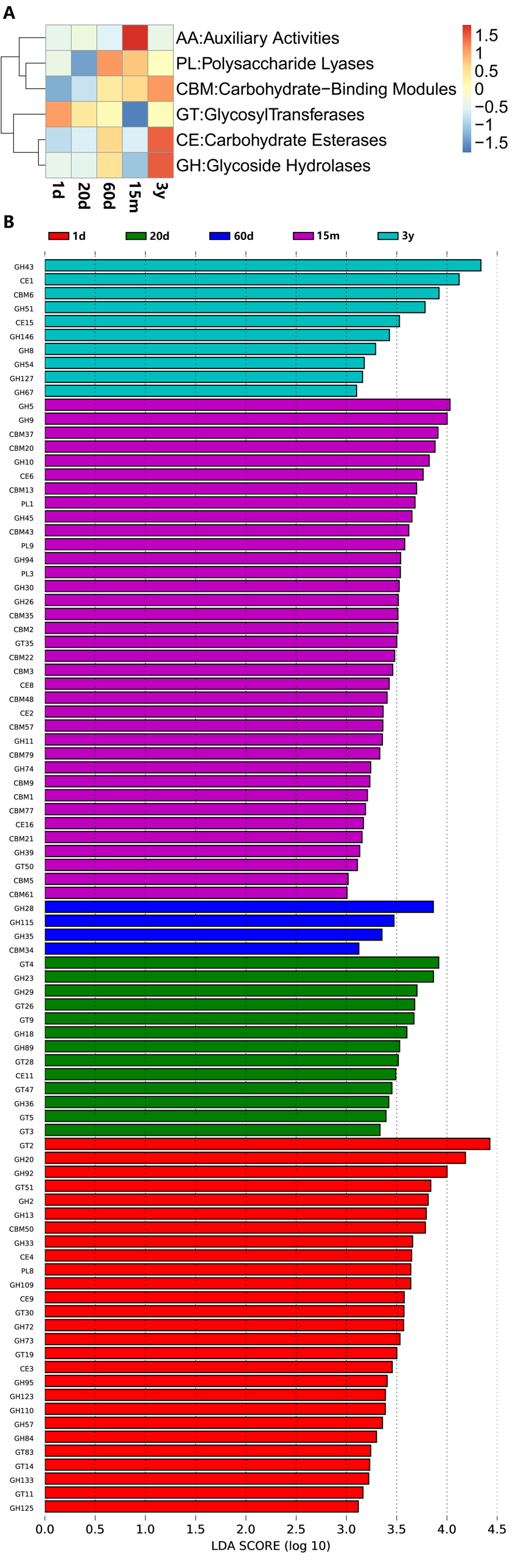

Supplement: Supplementary file 1 [file animals-14-01365-s001.zip › Figure S5-Heapmap and LEfSe analysis of CAZy enzymes.jpg]

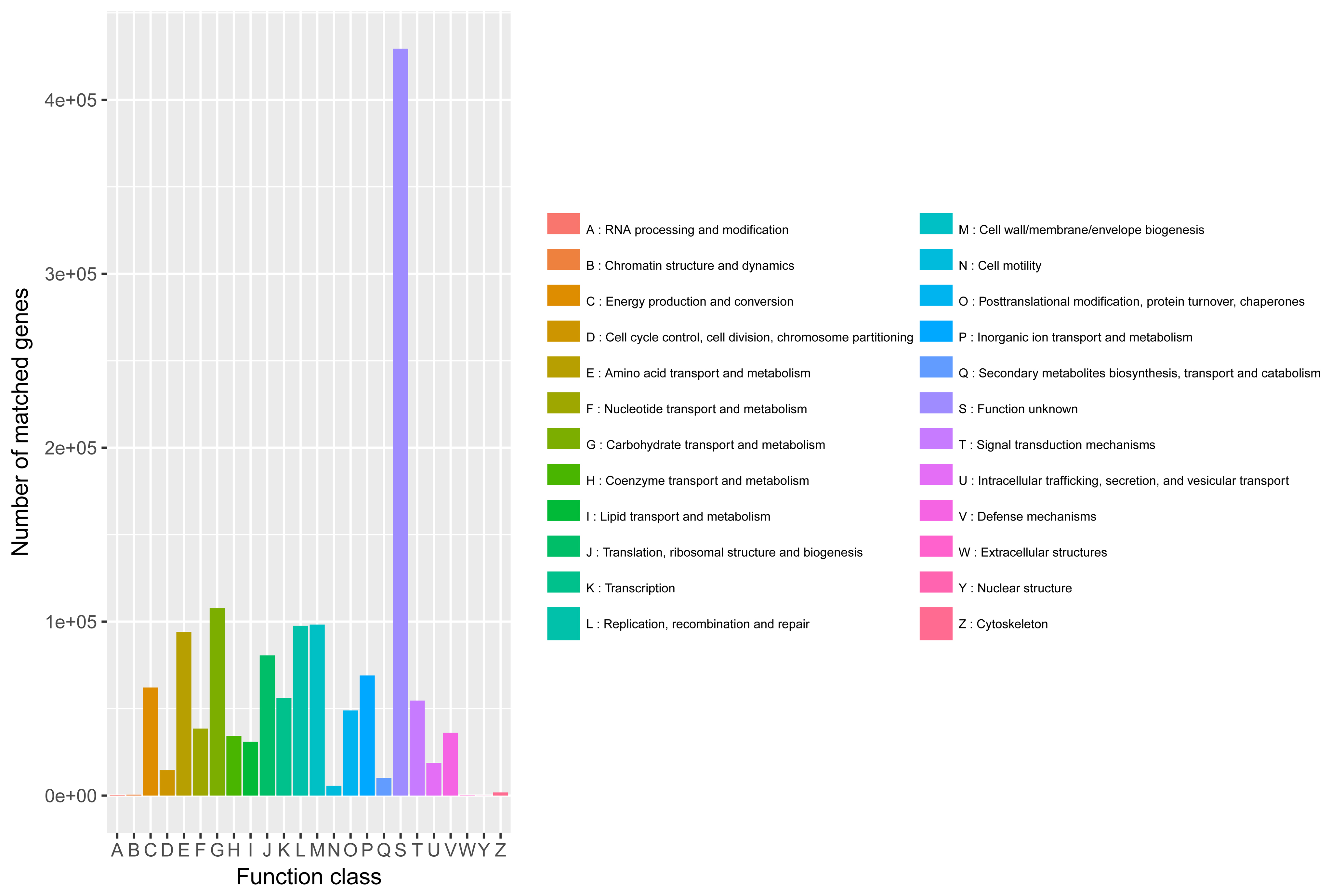

Supplement: Supplementary file 1 [file animals-14-01365-s001.zip › Figure S6-Distribution of eggNOG functional annotation of identified genes from rumen microbiota of yaks.jpg]

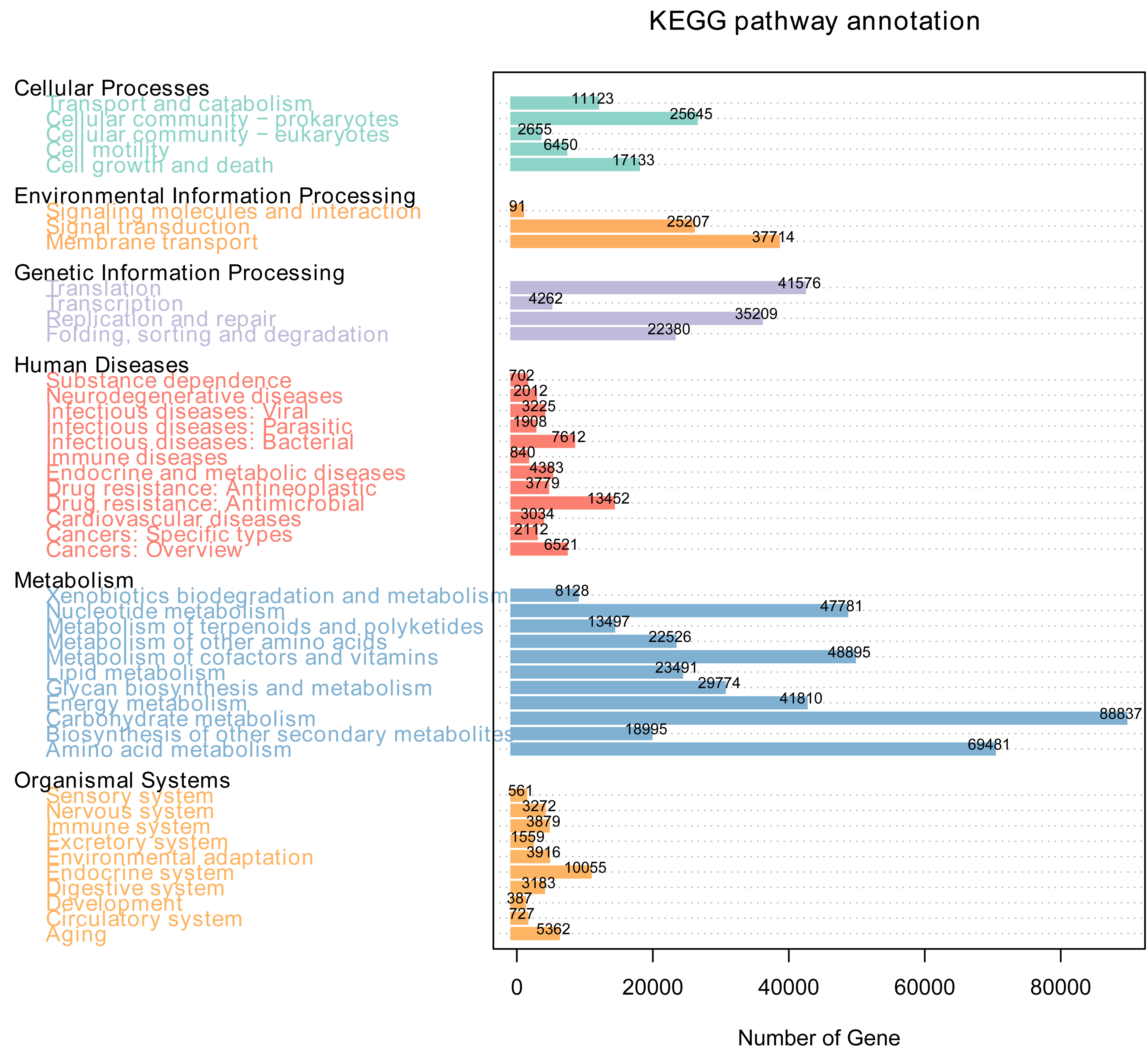

Supplement: Supplementary file 1 [file animals-14-01365-s001.zip › Figure S7-The number of genes annotated in KEGG pathways at level 1.jpg]
